# Automated Tobacco Cessation Intervention for Parents in Pediatric Primary Care: A Cluster-Randomized Clinical Trial

**DOI:** 10.1001/jamanetworkopen.2025.29384

**Published:** 2025-08-27

**Authors:** Emara Nabi-Burza, Brian P. Jenssen, Abra M. Jeffers, Janani Ramachandran, Jeritt G. Thayer, Bethany Hipple, Douglas E. Levy, Robert W. Grundmeier, Olivier Drouin, Mark Vangel, Nancy A. Rigotti, Tyra Bryant-Stephens, Ekaterina Nekrasova, Madeline McKnight, Jonathan P. Winickoff, Alexander G. Fiks

**Affiliations:** 1 Division of General Academic Pediatrics, Massachusetts General Hospital for Children, Boston; 2 Tobacco Research and Treatment Center, Massachusetts General Hospital, Boston; 3 Department of Pediatrics, Perelman School of Medicine, University of Pennsylvania, Philadelphia; 4 Clinical Futures, PolicyLab, and The Possibilities Project, Children’s Hospital of Philadelphia, Philadelphia, Pennsylvania; 5 Department of Biomedical and Health Informatics, Children’s Hospital of Philadelphia, Philadelphia, Pennsylvania; 6 Mongan Institute Health Policy Center, Massachusetts General Hospital, Boston, Massachusetts; 7 Harvard Medical School, Boston, Massachusetts; 8 Division of General Pediatrics, Department of Pediatrics, Centre Hospitalier Universitaire Sainte-Justine, Montréal, Quebec, Canada; 9 Department of Pediatrics, Faculty of Medicine, Université de Montréal, Montréal, Quebec, Canada; 10 Department of Social and Preventive Medicine, School of Public Health, Université de Montréal, Montréal, Quebec, Canada; 11 Department of Biostatistics, Massachusetts General Hospital, Boston; 12 University of North Carolina at Chapel Hill School of Medicine, Chapel Hill; 13 American Academy of Pediatrics, Julius B. Richmond Center of Excellence, Itasca, Illinois

## Abstract

**Importance:**

Treatment for tobacco use for parents in pediatric primary care settings is rarely provided but may support cessation and reduce childhood tobacco smoke exposure.

**Objective:**

To study the integration of the automated Clinical Effort Against Secondhand Smoke Exposure (eCEASE) tobacco cessation intervention into pediatric primary care via the electronic health record (EHR).

**Design, Setting, and Participants:**

This cluster-randomized clinical trial was conducted from July 16, 2021, to August 15, 2023, at 12 pediatric primary care practices in the Philadelphia, Pennsylvania, region. Participants included parents who used combusted tobacco in the past 7 days and attended a child’s preventive health care visit.

**Intervention:**

In all practices, household members completed EHR previsit questionnaires about tobacco use. Parents in intervention practices were proactively offered eCEASE (automated delivery of nicotine replacement therapy [NRT], quitline and/or SmokefreeTXT enrollment, and navigator support). Control practice parents received usual care.

**Main Outcomes and Measures:**

The primary outcome was biochemically confirmed 7-day abstinence from combusted tobacco use by parents at the 1-year follow-up. Secondary outcomes included NRT and quitline and/or SmokefreeTXT use and recent quit attempts. Changes in cigarettes per day and smoking frequency (daily or nondaily) from baseline to 1-year follow-up were also examined.

**Results:**

Of 817 enrolled smoking parents (672 [82.3%] female), 323 of 408 (79.2%) in the intervention arm (6 practices) and 326 of 409 (79.7%) in the control arm (6 practices) were mothers; mean (SD) age was 36.17 (8.67) years. The follow-up survey was completed by 367 of 408 parents (90.0%) in the intervention arm and 368 of 409 (90.0%) in the control arm. Biochemically confirmed 7-day abstinence rates were 34 of 408 (8.3%) in the intervention arm vs 26 of 409 (6.4%) in the control arm (adjusted odds ratio, 1.34; 95% CI, 0.79-2.29). Among those who completed follow-up, 177 of 367 (48.2%) in the intervention arm vs 59 of 368 (16.0%) in the control arm reported using NRT; 93 of 367 (22.8%) and 8 of 368 (2.2%), respectively, reported using quitline and/or SmokefreeTXT messaging; and 294 of 367 (80.1%) vs 258 of 368 (70.1%), respectively, reported a quit attempt in the last 3 months. Compared with control practices, intervention practices reported a greater reduction in the mean (SD) number of cigarettes smoked daily (−3.32 [5.39] vs −1.81 [5.84]) and a greater reduction in the mean (SD) percentage of daily smokers (−35.2% [2.6%] vs −25.8% [2.6%]).

**Conclusions and Relevance:**

In this cluster-randomized clinical trial of an automated intervention to treat parental tobacco use in pediatric practices, the intervention did not significantly improve the primary outcome of quit rate at 1 year. Findings in this trial demonstrated increased treatment engagement and reductions in cigarette consumption, but additional strategies are needed to improve quit rates.

**Trial Registration:**

ClinicalTrials.gov Identifier: NCT04974736

## Key Points

**Question:**

Does integrating an automated electronic health record–based tobacco cessation intervention in pediatric primary care practices reduce parental smoking?

**Findings:**

In a cluster-randomized clinical trial of 817 parents whose children were treated at 12 pediatric practices, the intervention, compared with usual care, did not significantly increase parental smoking cessation at 1 year (8.3% vs 6.4%), but it increased receipt of treatment and reduced cigarette consumption.

**Meaning:**

An automated electronic health record–based intervention improves parent tobacco treatment and reduces use; enhancements are needed to improve cessation outcomes.

## Teaser

This cluster-randomized clinical trial evaluates the implementation and effectiveness of an automated electronic health record–based tobacco cessation intervention targeting parents of children treated in a pediatric primary care setting.

## Introduction

A parent who quits smoking increases their life expectancy by as many as 10 years,^1^ eliminates future tobacco-related poor pregnancy outcomes,^2^ reduces the likelihood of their children becoming smokers,^3,4,5,6,7^ and decreases their children’s exposure to tobacco smoke.^8^ Lowering tobacco smoke exposure decreases the risk of tobacco smoke–related illnesses, resulting in fewer missed school days^9^ and less risk of developmental delays.^10,11^ Additionally, helping parents quit smoking may improve the financial resources of families^12^ and lower the risk of house fires.^13^ Parents who smoke are often medically underserved and may not have their own primary health care clinician but will see their child’s physician an average of 4 times per year.^14^

The Clinical Effort Against Secondhand Smoke Exposure (CEASE) intervention has been implemented in pediatric practices to address household smoking behavior in a routine and effective manner.^15,16,17,18,19^ Nevertheless, CEASE’s sustainability has been limited by the lack of systems-level integration and methods to facilitate automatic connection to external resources. The present study aimed to automate the screening and delivery of tobacco treatment in pediatric practices. This integration was achieved through the development of an innovative electronic health record (iEHR)–based approach that enhanced existing workflows in pediatric settings and offered tobacco cessation navigator support.^20,21^ The study evaluated the implementation and effectiveness of this intervention for treating parents who smoke.

## Methods

### Practice Enrollment and Randomization

We conducted a 2-arm parallel-group cluster-randomized clinical trial to compare the efficacy of the practice-based automated CEASE (eCEASE) intervention vs usual care in 12 pediatric practices in eastern Pennsylvania. Practices in the Pediatric Research Consortium^22^ primary care research network at Children’s Hospital of Philadelphia (CHOP) were recruited for the study. Practices were eligible if at least 10% of their pediatric patients had Medicaid coverage and they had a minimum of 40 daily visits. Eligible practices were paired based on size and estimated smoking rate, with 6 pairs selected to ensure sufficient size and representativeness. The study was initially planned with clinician-level randomization due to anticipated clinician autonomy in intervention delivery. However, randomization was ultimately conducted at the practice level due to EHR deployment constraints and concerns about contamination within practices. Within each pair, practices were randomly assigned to either the intervention or control group using computer-generated allocation. The statistician (M.V.) remained blinded throughout final analysis, but practices could not be blinded to group assignment. The study protocol was approved by the institutional review boards at CHOP and Massachusetts General Hospital. This study followed the Consolidated Standards of Reporting Trials (CONSORT) reporting guidelines. The trial protocol is found in Supplement 1.

### Sample Size Calculation

Sample size calculations were based on estimates of validated 12-month abstinence rates using data from prior studies assuming 13.8% in the intervention group and 7.0% in the usual care control group.^19,23,24^ These estimates were also based on the worst-case scenario where all enrolled parents lost to follow-up are assumed to be smokers at the 12-month follow-up. The sample size was determined assuming 20 clinician clusters per arm. The intraclass correlation coefficient of 0.014 was calculated from a previous study.^23^ By enrolling approximately 400 per arm, we expected to have 80% power for the primary outcome.

### Parent Eligibility and Enrollment

Prior to a well-child visit, parents or legal guardians (henceforth parents) were asked to complete an electronic screening questionnaire about tobacco use via patient portal (MyChart; Epic Systems Corporation) or tablet at the clinic. Current combusted tobacco use by a parent was defined as answering “yes” to either of 2 screening questions: “Have you smoked a cigarette, even a puff, in the past 7 days?” or “Have you smoked any other tobacco product (cigars like Black and Mild, hookah), even a puff, in the past 7 days?”

Research assistants reviewed the previsit questionnaires and contacted eligible parents in person at the visit. Due to the COVID-19 pandemic, screening and enrollment procedures were subsequently adjusted to remote strategies (telephone, text, or email) after the visit. Parents who did not speak English, had no telephone, or were already enrolled in the study were excluded. Eligible parents were offered enrollment in the study, completed an electronic signed informed consent form, and received $20 for completing a baseline survey. Enrollment continued until each arm included approximately 400 parents. Twelve months after enrollment, parents received $40 for completing a follow-up survey in person or by telephone, text, or email. The Figure shows the CONSORT diagram.

**Figure.**
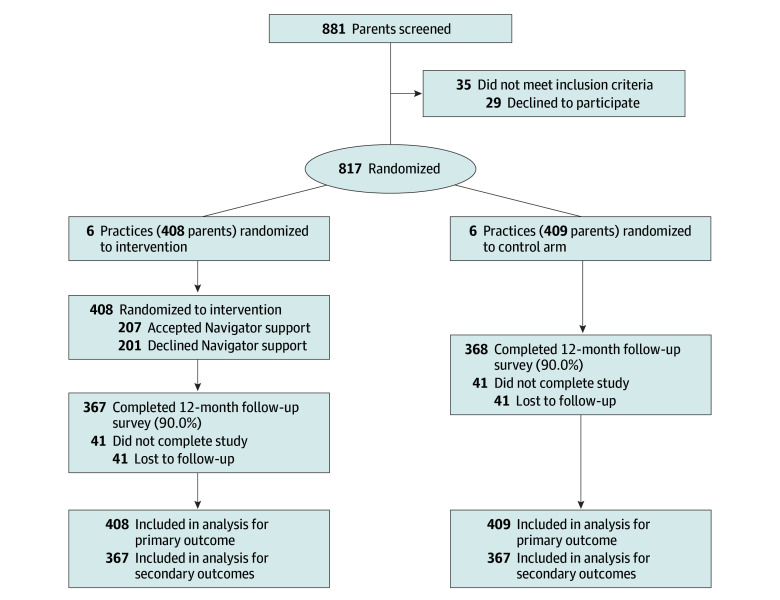
Study Flow Diagram

### Intervention

The eCEASE intervention aimed to support parental smoking cessation via an electronic health record platform plus an offer of tobacco cessation navigator support at enrollment. The platform delivered evidence-based smoking cessation strategies, including household tobacco use screening, automatic nicotine replacement therapy (NRT) provision, and enrollment in evidence-based cessation services (quitline and the SmokefreeTXT mobile messaging service).^20,21^

Intervention components consisted of the following:

1. Identification of parents who smoke tobacco: Parents completed the questionnaire via the patient portal online before or in person during the visit via tablet.
2. Counseling: The questionnaire displayed motivational smoking cessation messages^25^ to encourage treatment acceptance.
3. Automated NRT prescription and delivery: For parents who smoked and did not opt out, the electronic health record platform autogenerated NRT prescriptions for patches and gum with details prepopulated from the questionnaire. It was electronically sent to a local pharmacy, which delivered the NRT to parents within 48 hours.
4. Automated quitline and SmokefreeTXT enrollment: Unless they opted out, the electronic health record platform automatically enrolled parents who smoke in the state’s free quitline and the National Cancer Institute’s SmokefreeTXT program. SmokefreeTXT sent texts immediately after enrollment, while the quitline contacted parents within 48 hours.
5. Community health navigator support: At enrollment, a research assistant offered community health navigator support, which provided smoking cessation assistance through home visits and/or telephone (primarily by telephone due to COVID-19). Support included coaching, facilitating medication access, and connecting parents to services.
6. Pediatric practice staff training: All office staff (clinical and nonclinical) in the intervention practices were trained in using the platform to support families who smoke. Training included a presentation for the entire office, direct in-person training, and ongoing support.

### Usual-Care Control

The usual-care control arm received CHOP’s standard care, including routine assessment and guidance per institutional guidelines. Prior work found that this rarely involved parent counseling on smoking, and parents were almost never prescribed medication to promote cessation.^26^

### Measures and Outcomes

The baseline enrollment survey collected parents’ self-reported demographic characteristics (age, sex, race and ethnicity [as required by the National Institutes of Health], insurance status, and level of education) and age of the youngest child at the visit. Parents were also asked about their smoking habits (“Do you now smoke cigarettes every day or some days?”), number of cigarettes smoked per day, and their readiness to quit. At 1-year follow-up, parents were asked again about current tobacco use, including frequency and quantity. They were also asked about receiving tobacco cessation assistance at the pediatric practice in the past year and services they used (“Have you talked about smoking with someone at a quitline?” “Have you used SmokefreeTXT service since your enrollment?” and “Have you used any nicotine replacement medicine such as nicotine gum, patch, or lozenge, etc, since your enrollment?”).

The primary outcome of the study was parents’ biochemically confirmed past 7-day tobacco abstinence at 1-year follow up. Parents who self-reported abstinence of all combusted tobacco products were invited to submit a saliva sample for cotinine testing, a nicotine metabolite.^27^ Parents reporting current use of NRT or e-cigarettes or had a cotinine level of 10 ng/mL or greater (to convert to nmol/L, multiply by 5.68) were given the opportunity to submit carbon monoxide breath levels (<5 ppm)^28^ to confirm self-reported cessation. Parents with missing follow-up survey or biochemical validation were considered smokers.

Secondary outcomes were rates of use of NRT, use of quitline and/or SmokefreeTXT, and quit attempts in the last 3 months. We also examined changes in cigarettes per day and smoking frequency (daily vs nondaily) from baseline to 1-year follow-up.

### Statistical Analysis

Consistent with the practice-level randomization, we clustered by practice for the analysis. We included random effects to model the randomization process: a random effect for each practice pair, with a nested between-arm random effect. We used generalized linear mixed-effects models to compare the primary and secondary outcomes between arms. All analyses were performed at the end of data collection in R, version 4.4.2,^29^ using the lme4 and lmerTest packages.^30,31^ Hypotheses were tested using likelihood ratio χ^2^ tests, except for risk differences between arms, for which we applied the delta method. Difference-in-difference analyses from baseline to 12-month follow-up were conducted to evaluate the effect of the intervention on changes in mean cigarettes per day and daily smoking rates. We used Bonferroni adjustment to control the familywise error rate at 5% across tests with 5 secondary outcomes. Within the intervention arm, an exploratory analysis compared those who accepted navigator support with those who did not. An additional logistic regression analysis explored factors associated with the use of NRT. Two-sided *P* < .05 indicated statistical significance.

## Results

Parents completed the baseline and 1-year follow-up surveys between July 16, 2021, and August 15, 2023. A total of 817 parents who smoked were enrolled in the study (672 female [82.3%], 143 male [17.5%], and 2 who did not wish to share [0.2%]; mean [SD] age, 36.17 [8.67] years), including 408 in the intervention (6 practices) and 409 in the control (6 practices) arms. A total of 68 parents (8.3%) were Hispanic; 3 (0.4%), non-Hispanic Alaska Native, American Indian, or Native Hawaiian; 20 (2.4%), non-Hispanic Asian; 330 (40.4%), non-Hispanic Black; 356 (43.6%), non-Hispanic White; and 40 (4.9%), non-Hispanic multiracial. Table 1 presents participant characteristics. Eligibility, consent, and enrollment rates were similar across study arms, indicating no bias. The arms were overall balanced in demographic characteristics, except for slight differences in the proportions of Non-Hispanic Black and White participants. EHR visit data showed children younger than 2 years had a mean (SD) of 7.0 (3.8) visits per year; those aged 2 to 4 years, 3.1 (2.2) visits per year; and those 5 years or older, 1.5 (2.1) visits per year.

**Table 1.** Characteristics of Parents Who Smoked Enrolled in the Study at Baseline

Characteristic	Parent group, No. (%)	
	Control (n = 409)	Intervention (n = 408)
Age, mean (SD), y	35.9 (8.5)	36.4 (8.9)
Sex		
Female	337 (82.4)	335 (82.1)
Male	71 (17.4)	72 (17.6)
Do not wish to share	1 (0.2)	1 (0.2)
Relationship to the child		
Mother	326 (79.7)	323 (79.2)
Father	68 (16.6)	71 (17.4)
Other	15 (3.7)	14 (3.4)
If other, legal guardian	14 (3.4)	14 (3.4)
Race and ethnicity		
Hispanic, Latino or Spanish origin	28 (6.8)	40 (9.8)
Non-Hispanic Alaska Native, American Indian, or Native Hawaiian only	1 (0.2)	2 (0.5)
Non-Hispanic Asian only	3 (0.7)	17 (4.2)
Non-Hispanic Black or African American only	172 (42.1)	158 (38.7)
Non-Hispanic White only	189 (46.2)	167 (40.9)
Non-Hispanic multiracial	16 (3.9)	24 (5.9)
Educational attainment		
Did not finish high school	35 (8.6)	40 (9.8)
High school graduate	193 (47.2)	175 (42.9)
Some college	124 (30.3)	113 (27.7)
College graduate	57 (13.9)	80 (19.6)
Age of youngest child seen at pediatric practice		
<1 y	77 (18.8)	80 (19.6)
1-4 y	145 (35.5)	135 (33.1)
5-9 y	88 (21.5)	98 (24.0)
10-14 y	70 (17.1)	75 (18.4)
≥15	29 (7.1)	20 (4.9)
Parents’ insurance coverage		
Insurance from employer	102 (24.9)	104 (25.5)
Purchased directly from insurer	15 (3.7)	11 (2.7)
Medicaid	253 (61.9)	260 (63.7)
Tricare or other military health care	1 (0.2)	4 (1.0)
Not insured	13 (3.2)	12 (2.9)
Other	25 (6.1)	17 (4.2)
No. of cigarettes/d, mean (SD)	8.3 (5.8)	8.2 (5.9)
Plan to quit		
Next 6 mo	305 (74.6)	318 (77.9)
Next 30 d (among those who plan to quit in the next 6 mo)	144 (47.2)	215 (67.6)
Quit attempt in the past 3 mo	173 (42.3)	176 (43.1)

### Biochemically Confirmed Quit Rate

Of the enrolled parents, 367 (90.0%) in the intervention arm and 368 (90.0%) in the control arm completed the 12-month follow-up survey. Seven-day abstinence was self-reported by 89 parents (21.8%) in the intervention arm vs 69 (16.9%) in the control arm (risk difference, 4.7; 95% CI, –0.7 to 10.2). Of these, 40 parents (44.9%) in the intervention and 31 (44.9%) in the control arms returned the cotinine or carbon monoxide samples. Biochemically confirmed abstinence rate was 34 (8.3%) in the intervention arm and 26 (6.4%) in the control arm (risk difference, 2.0; 95% CI, −1.6 to 5.6). After adjusting for parent age, insurance type, and smoking intensity, the adjusted odds ratio (AOR) for biochemically confirmed quitting in the intervention vs control practices was 1.34 (95% CI, 0.79-2.29), assuming all missing samples were smokers. We also conducted sensitivity analyses assuming missing samples reflected observed confirmation rates (OR, 1.38; 95% CI, 0.94-2.05) and assuming all self-reported quitters had quit (OR, 1.38; 95% CI, 0.95-2.01). In all analysis, quit rates consistently favored the intervention but without statistical significance. Among those who self-reported quitting but did not return the cotinine test, there were 49 cases in the intervention arm and 38 in the control arm. Reclassifying 8 of the 49 intervention cases as nonsmokers while keeping all control cases unchanged would make the primary outcome statistically significant (OR, 1.69; 95% CI, 1.02-2.81; *P* = .04, Fisher exact test). To address missing biochemical verification, we conducted a sensitivity analysis using a composite outcome of self-reported 7-day abstinence plus either cotinine-confirmed quitting or ongoing NRT use (as NRT use may increase cotinine levels). Using this definition, 37 (9.1%) in the intervention and 20 (4.9%) in the control arm met cessation criteria. Odds of quitting were significantly higher in the intervention arm (OR, 1.94; 95% CI, 1.11-3.40).

### Smoking Frequency and Quantity

There was a greater reduction in the mean (SD) number of cigarettes smoked per day in the intervention arm (−3.32 [5.39] cigarettes/d) compared with the control arm (−1.81 [5.84] cigarettes/d) (Table 2). A significantly higher proportion of parents in the intervention arm transitioned from daily to nondaily smoking compared with the control arm (mean [SE], −35.2% [2.6%] vs −25.8% [2.6%]). Within the intervention arm, parents who accepted the navigator support, compared with those who did not, reduced the mean [SD] number of cigarettes smoked per day (−4.62 [0.38] vs −1.93 [0.39] cigarettes/d), and were more likely to transition from daily to nondaily smokers (mean [SE], −44.7% [3.8%] vs −24.8% [3.9%]).

**Table 2.** Behavioral Outcomes at 12-Month Follow-Up

Behavior	Control arm			Intervention arm			Difference in differences, mean change (95% CI)
	Baseline (n = 409)	12-mo Follow-up (n = 368)	Mean change from baseline to 12 mo	Baseline (n = 408)	12-mo Follow-up (n = 367)	Mean change from baseline to 12 mo	
Mean cigarettes/d (SD)	8.32 (5.83)	6.53 (6.69)	−1.81 (SD, 5.84)	8.15 (5.92)	4.94 (5.50)	−3.32 (SD, 5.39)	−1.51 (−2.28 to −0.74)
Daily smokers, No. (%)	348 (85.1)	220 (59.8)	−25.8% (SE, 2.6%)	345 (84.6)	182 (49.6)	−35.2% (SE, 2.6%)	−9.4% (−16.6 to −2.2)

### Engagement With Tobacco Cessation Services or Treatments, Quit Attempts, and Smoking or Vaping in Homes and Cars

Parents in the intervention arm were more likely to report using cessation medications and/or services (Table 3). Among those who completed the follow-up, 177 (48.2%) in the intervention group and 59 (16.0%) in the control group (*P* < .001) reported using NRT since enrollment; 93 (25.3%) in the intervention group and 8 (2.2%) in the control group (*P* < .001) reported using the quitline or the SmokefreeTXT service; and 294 of 367 (80.1%) vs 258 of 368 (70.1%), respectively, reported a quit attempt in the last 3 months. Within the intervention arm, parents who accepted navigator support were more likely to use NRT than those who did not (121 of 188 [64.4%] vs 55 of 178 [30.9%]). Quit attempts in the past 3 months and smoking or vaping in homes and cars did not differ significantly between arms.

**Table 3.** Secondary Outcomes

Outcome	Study arm, No. (%) of parents		Bonferroni-adjusted *P* value
	Control (n = 368)	Intervention (n = 367)	
Rates of use of pharmacotherapy or services			
Used NRT since enrollment	59 (16.0)	177 (48.2)	<.001
Used quitline or SmokefreeTXT	8 (2.2)	93 (25.3)	<.001
Quit attempts in the past 3 mo	258 (70.1)	(80.1)	.008
Smoking or vaping in homes	137 (37.2)	124 (33.8)	>.99
Smoking or vaping in cars	141 (38.3)	127 (34.6)	>.99

Abbreviation: NRT, nicotine replacement therapy.

### Additional Analysis

In an exploratory analysis looking at factors associated with NRT use in the overall sample, we modeled a fixed-effects generalized linear model with use of NRT as the binary dependent variable on the interaction of having Medicaid insurance and being in the intervention arm, while controlling for parent age, race (categorized as Black, White, or other), and educational attainment (any college vs no college). We found that receiving the intervention in the non-Medicaid group was associated with increased NRT use compared with being in the control group, keeping other covariates constant (AOR, 2.15; 95% CI, 1.26-3.73). Parents with Medicaid in the intervention arm had 8 times greater odds of using NRT than those with Medicaid in the control arm (AOR, 8.00; 95% CI, 5.00-12.80) (Table 4).

**Table 4.** Exploratory Multivariable Logistic Regression Results: Factors Associated With NRT Use

Characteristic	AOR (95% CI)
Demographics	
Parent age (per y increase)	1.03 (1.01-1.054)
Race and ethnicity	
Black	1 [Reference]
White	1.01 (0.70-1.47)
Other	1.36 (0.80-2.33)
Educational attainment	
Any college	0.95 (0.67-1.35)
No college	1 [Reference]
Control arm	
Medicaid	0.57 (0.32-1.03)
Non-Medicaid	1 [Reference]
Non-Medicaid	
Intervention arm	2.15 (1.26-3.73)
Control arm	1 [Reference]
Medicaid	
Intervention arm	8.00 (4.98-12.80)
Control arm	1 [Reference]

Abbreviations: AOR, adjusted odds ratio; NRT, nicotine replacement therapy.

## Discussion

In this cluster-randomized clinical trial of 12 pediatric practices within a pediatric primary care network, we found that the biochemically confirmed quit rate (primary outcome) in the intervention arm (8.3%) was not significantly higher than that in the control arm (6.4%). However, integrating a parental smoking cessation intervention through an EHR platform resulted in significantly increased tobacco treatment delivery, use of cessation services by parents, and reduced parental cigarette consumption during 1 year.

There are several possible explanations for the lack of a significant difference in biochemically confirmed quit rates between study arms. Navigator support was only provided during enrollment, when parents might not have been ready to quit smoking—a crucial factor that changes through stages^32,33,34^—and might have required ongoing assistance. Second, while the intervention offered 3 evidence-based treatments, it did not offer the full range of US Food and Drug Administration–approved medications such as bupropion and varenicline. Incorporating more effective medications paired with cognitive behavioral therapy might have improved quit rates. Third, while the automated screening and treatment delivery reduced clinician burden, the intervention might have been too hands off on the clinician side, potentially missing the opportunity for personalized engagement and support that could have motivated and guided parents through cessation. Enhancing clinician engagement through improved compensation for smoking cessation counseling and educational support might have strengthened the effectiveness of the eCEASE intervention and improved quit rates.

Although the intervention did not result in a statistically significant increase in cessation rates after 1 year, it did reduce parents’ daily cigarette consumption. Evidence suggests that reducing smoking can promote cessation.^35^ In a survey of 1000 current daily cigarette smokers,^36^ nearly half (44%) planning to quit within the next year preferred gradual cessation, and most (68%) were open to using products or medications to reduce smoking. This aligns with our findings, where parents actively used available treatments and services to cut down smoking, even though they may not have quit. Even though higher rates of quitting were not achieved, the reduction in smoking represents a meaningful intermediate outcome, reducing tobacco smoke exposure of children, improving health by reducing smoking-related risks, and contributing to long-term public health goals by promoting gradual progress toward cessation.

Parents in intervention practices were significantly more likely to use NRT and other cessation resources than those in the control practices. Research shows that use of evidence-based treatments increases quit rates.^37^ This highlights the potential of automated interventions in pediatric care to expand access for those who would otherwise not get them. To further improve long-term quitting success, we plan to offer the most effective treatments, including bupropion and varenicline.^38,39^ eCEASE is currently housed within a widely used EHR platform (Epic Systems Corporation), facilitating scaling to other pediatric primary care settings around the nation.

For those parents who opted for the navigator support at the time of consent, we found higher rates of use of NRT, as well as a reduction in their smoking frequency and amount compared with those who did not opt for navigator support. A previous study suggests that a smoking cessation navigator can significantly enhance the cessation process by providing personalized guidance, support, and expert advice on use and duration of pharmacotherapy.^24^ Taken together, these findings suggest the importance of navigator support in enhancing smoking cessation efforts among parents. However, the findings from our study should be viewed as exploratory because the smokers who accepted navigator support to help them quit smoking might have been more motivated to quit smoking.

We also found increased use of NRT among parents with Medicaid in the intervention arm, underscoring the critical role of programs like eCEASE in promoting equitable access to smoking cessation services across diverse socioeconomic groups. By ensuring that effective cessation resources are accessible to all parents, regardless of their financial situation, such programs can reduce health disparities and improve family health outcomes.

### Limitations

This study has some limitations. It was conducted in a single health system in Philadelphia. While the practices were diverse, this may limit the generalizability of the results. Research assistants were not blinded to study arm assignment, but rigorous training was provided to ensure consistent data collection across groups. The eCEASE intervention provided navigator support only at enrollment, which may not have aligned with some parents’ readiness to quit. A more flexible or repeated engagement approach could enhance long-term cessation outcomes. Excluding non–English-speaking parents in this trial may limit generalizability to English-speaking populations. Last, the sample size was powered assuming clinician-level clustering, while randomization occurred at the practice level. Accordingly, our primary analysis accounted for clustering by practice. We also conducted a secondary analysis with clustering at the clinician level, consistent with the original power calculation, which yielded a similarly nonsignificant result for the primary outcome.

## Conclusions

In this cluster-randomized clinical trial, an automated parental tobacco use intervention in pediatric primary care did not significantly improve the primary outcome of quit rates at 1 year. The trial showed significantly increased treatment engagement and reductions in cigarette use, with nearly half of parents in the intervention arm using NRT and 35.2% reducing daily smoking compared with 16.0% using NRT and 25.8% reducing daily smoking in the control arm. These results suggest that additional strategies are needed to achieve meaningful improvements in quit rates and that such strategies may influence behavior when deployed through this platform.

## Notes

Supplement 1.
Trial Protocol

Supplement 2.
Data Sharing Statement
