# Supplementary material for: Automated Tobacco Cessation Intervention for Parents in Pediatric Primary Care: A Cluster-Randomized Clinical Trial
Source: JAMA Netw Open. 2025 Aug 27;8(8):e2529384. doi: 10.1001/jamanetworkopen.2025.29384 (PMC12391990; doi:10.1001/jamanetworkopen.2025.29384)
Supplement: Supplement 1. — Trial Protocol [file jamanetwopen-e2529384-s001.pdf]

Title:	**Electronic Pediatric Office Systems to Support Treatment for Parental Tobacco Use**
Short Title	eCEASE
Drug or Device Name(s):	N/A
NCT Number:	NCT04974736
Sponsor:	NIH
eIRB Number:	IRB 20-018146

Protocol Date: 04/01/2025

Amendment 1 Date: 06/21/2021	Amendment 2 Date (Staff Change): 07/07/2021
Amendment 3 Date (Staff Change): 07/14/2021	Amendment 4 Date (Staff Change): 07/23/2021
Amendment 5 Date: 9/29/2021	Amendment 6 Date (Staff change): 10/04/2021
Amendment 7 Date (Staff change): 12/16/2021	Amendment 8 Date (Staff change): 1/14/2022
Amendment 9 Date: 1/19/2022.	Amendment 10 (Staff change) Date: 2/1/2022
Amendment 11 Date: 2/16/2022	Amendment 12 (Staff change) Date: 2/25/2022
Amendment 13 (staff change) Date: 3/15/2022	Amendment 14 Date: 6/2/2022
Amendment 15 (staff change) Date: 6/4/2022	Amendment 16 (staff change): 06/13/2022
Amendment 17 Date: 6/13/2022	Amendment 18 (staff change): 06/23/2022
Amendment 19 (staff removal) Date: 07/05/2022	Amendment 20: 08/16/2022
Amendment 21 (staff change) Date: 10/06/2022	Amendment 22 (staff change): 12/05/2022
Amendment 23 (staff removal) Date: 01/12/2023	Amendment 24 (staff change): 02/03/2023
Amendment 25 Date: 2/24/2023	Amendment 26 (staff change): 04/03/2023
Amendment 27: Date: 05/02/2023	Amendment 28: (staff removal): 06/07/2023
Amendment 29: (staff removal) 04/05/2024	Amendment 30: (staff removal): 03/20/2025
Amendment 31: 04/01/2025	

Sponsor: National Institutes of Health, NCI
6701 Rockledge Drive MSC 7768
Bethesda MD 20892-7768

TABLE OF CONTENTS

TABLE OF CONTENTS 2

ABBREVIATIONS AND DEFINITIONS OF TERMS 5

ABSTRACT 6

PROTOCOL SYNOPSIS 8

TABLE 1: SCHEDULE OF STUDY PROCEDURES 12

1 BACKGROUND INFORMATION AND RATIONALE 14

1.1 INTRODUCTION 14

1.2 NAME AND DESCRIPTION OF INVESTIGATIONAL PRODUCT OR INTERVENTION 15

1.3 RELEVANT LITERATURE AND DATA 15

1.4 COMPLIANCE STATEMENT 17

2 STUDY OBJECTIVES 17

2.1 PRIMARY OBJECTIVE 17

2.2 SECONDARY OBJECTIVES. 17

3 INVESTIGATIONAL PLAN 19

3.1 GENERAL SCHEMA OF STUDY DESIGN 19

3.1.1 PRACTICE SELECTION AND RANDOMIZATION 19

3.1.2 PARENT SCREENING & ENROLLMENT 19

3.1.3 INTERVENTION 20

3.1.4 FOLLOW-UP 20

3.2 PRACTICE SELECTION, RANDOMIZATION AND BLINDING 21

3.3 STUDY DURATION, ENROLLMENT AND NUMBER OF SITES 21

3.3.1 DURATION OF STUDY PARTICIPATION 21

3.3.2 TOTAL NUMBER OF STUDY SITES/TOTAL NUMBER OF SUBJECTS PROJECTED 22

3.4 STUDY POPULATION 22

3.4.1 INCLUSION CRITERIA 22

3.4.2 EXCLUSION CRITERIA 23

4 STUDY PROCEDURES 23

4.1 SCREENING & ENROLLMENT 23

4.2 INTERVENTION 27

4.2.1 IEHR 27

4.2.2 COMMUNITY HEALTH NAVIGATORS (CHNS) 28

4.3 FOLLOW-UP 29

4.3.1 PARENT FOLLOW-UP 29

4.3.2 ONGOING EHR DOCUMENTATION & EVALUATION 31

4.3.3 PRACTICE CLINICIANS AND STAFF QUALITATIVE INTERVIEWS 31

4.4 SUBJECT COMPLETION/WITHDRAWAL 32
4.4.1 EARLY TERMINATION STUDY VISIT 32
5 STUDY EVALUATIONS AND MEASUREMENTS 32
5.1 SCREENING AND MONITORING EVALUATIONS AND MEASUREMENTS 32
5.1.1 MEDICAL RECORD REVIEW 32
5.1.2 LABORATORY EVALUATIONS 33
5.2 EFFICACY EVALUATIONS 35
5.2.1 DIAGNOSTIC TESTS, SCALES, MEASURES, ETC. 37
5.3 SAFETY EVALUATION 37
6 STATISTICAL CONSIDERATIONS 37
6.1 PRIMARY ENDPOINT 37
6.2 SECONDARY ENDPOINTS 37
6.3 STATISTICAL METHODS 37
6.3.1 BASELINE DATA 38
6.3.2 SAFETY ANALYSIS 38
6.4 SAMPLE SIZE AND POWER 38
7 STUDY INTERVENTION 39
7.1 DESCRIPTION 39
7.1.1 TREATMENT COMPLIANCE AND ADHERENCE 39
8 SAFETY MANAGEMENT 39
8.1 CLINICAL ADVERSE EVENTS 39
8.2 ADVERSE EVENT REPORTING 39
9 STUDY ADMINISTRATION 39
9.1 TREATMENT ASSIGNMENT METHODS 39
9.1.1 RANDOMIZATION 40
9.1.2 BLINDING 40
9.1.3 UNBLINDING 40
9.2 DATA COLLECTION AND MANAGEMENT 40
9.2.1 BASELINE SURVEY AND 12-MONTHS FOLLOW-UP DATA 41
9.2.2 DATA SHARING WITH THE QUITLINE 41
9.2.3 DATA SHARING WITH THE SMOKEFREETXT 42
9.2.4 DATA SHARING WITH THE BRIGHTMEDICAL 42
9.2.5 DATA TRANSFER OF THE EHR EXTRACTIONS BETWEEN CHOP AND MGH 42
9.2.6 DATA SHARING WITH THE ADA TRANSCRIPTION 43
9.2.7 CONFIDENTIALITY 43
9.2.8 SECURITY 46
9.2.9 ANONYMIZATION, DE-IDENTIFICATION OR DESTRUCTION. 46

9.3 CONFIDENTIALITY **47**

9.4 REGULATORY AND ETHICAL CONSIDERATIONS **47**

9.4.1 DATA AND SAFETY MONITORING PLAN **47**

9.4.2 RISK ASSESSMENT **48**

9.4.3 POTENTIAL BENEFITS OF TRIAL PARTICIPATION **50**

9.4.4 RISK-BENEFIT ASSESSMENT **50**

9.5 RECRUITMENT STRATEGY **51**

9.6 INFORMED CONSENT/ASSENT AND HIPAA AUTHORIZATION **52**

9.6.1 WAIVER OF CONSENT **53**

9.6.2 WAIVER OF HIPAA AUTHORIZATION **53**

9.7 PAYMENT TO SUBJECTS/FAMILIES **53**

9.7.1 PAYMENTS TO PARENT FOR TIME AND INCONVENIENCE (I.E., COMPENSATION) **53**

9.7.2 PAYMENTS TO PRACTICES FOR TIME, EFFORT AND INCONVENIENCE (I.E., COMPENSATION) **54**

9.7.3 PAYMENTS TO CLINICIANS AND STAFF FOR TIME, EFFORT AND INCONVENIENCE (I.E., COMPENSATION) **54**

10 PUBLICATION **54**

11 REFERENCES **54**

APPENDIX **59**

ABBREVIATIONS AND DEFINITIONS OF TERMS

CEASE	Clinical Effort Against Secondhand Smoke Exposure
AAP	American Academy of Pediatrics
CHN	Community Health Navigators
EHR	Electronic Health Record
iEHR	Innovative Electronic Health Record
TSE	Tobacco Smoke Exposure
SHS	Secondhand Smoke
NRT	Nicotine Replacement Treatment

ABSTRACT

Context: When parents quit smoking, their life expectancy is increased, their children have lower odds of becoming adult smokers, and their children may no longer be exposed to high levels of tobacco smoke, decreasing the odds of tobacco smoke exposure related health problems. Parents who smoke are often medically underserved and visit their child's healthcare clinician more often than they see their own clinician if they even have one. Routinely delivered tobacco control to parents in child healthcare settings would benefit the overall health of the nation.

Objectives: To compare parents' combusted tobacco quit rates, and adoption of tobacco free behaviors between the two arms (iEHR + Navigator vs. usual care control)

Study Design: Cluster randomized controlled trial of child healthcare practices with at least 4 practices each randomized to iEHR + Navigator or usual care control arms.

Setting/Participants: We will recruit at least 8 primary care pediatric practices in a large primary care practice network, The Pediatrics Research Consortium (PeRC), at CHOP.

In each arm to collect data related to the primary outcome, we will enroll in the longitudinal cohort approximately 400 parents or legal guardians who smoke, who are present at the visit, and whose child is seen by a child healthcare clinician in a participating practice. We may also add additional practices to maximize the likelihood of meeting the recruitment goal of 400 parents enrolled in each arm.

Study Interventions and Measures: The iEHR + Navigator intervention includes an innovative EHR platform (iEHR) with the addition of community health navigators (CHNs). iEHR consists of an electronic platform for smoking screening and automated smoking cessation support for parents who smoke. iEHR will include automated EHR-generated Nicotine replacement treatment (NRT) prescriptions, automated enrollment in the free state tobacco Quitline, and EHR prompts for the clinician to deliver a brief motivational message. CHN's will work with offices to provide customized cessation support to household tobacco users through phone follow-up, ensuring access to medications and services, and/or home visits, depending on the needs of each family and as study resources allow.

Primary outcome: Parental combusted tobacco quit rate at 1 year, as assessed by validated surveys, biochemically-confirmed at 12-months compared between iEHR + Navigator vs. usual care control.

Secondary Outcomes:

1. To compare parents' self-reported adoption of tobacco free behaviors (quit attempts, use of pharmacotherapy, use of Quitlines and institution of smoking and vaping bans in their homes and cars) between the iEHR + Navigator and usual care arms
2. To establish the incremental cost per quit of the iEHR + Navigator vs. usual care arms
3. To assess the implementation and sustainability of the intervention in the iEHR + Navigator arm (NRT prescription and Quitline referrals as assessed by the parental report around 12-months, the screening survey conducted at check-in, and EHR documentation

of screening and services delivery fidelity, before and immediately following implementation, 6, 12, 18, 24, 30, and 36 months).

PROTOCOL SYNOPSIS

**Study Title**	**Electronic Pediatric Office Systems to Support Treatment for Parental Tobacco Use**
**Funder**	NIH
**Study Rationale**	**Rationale for iEHR + Navigator.** The Clinical Effort Against Secondhand Smoke Exposure (CEASE) intervention has been successfully developed and implemented to train child healthcare clinicians to address household smoking behavior and promote smoke-free and e-cigarette free home and car rules in a routine and effective manner.[1,2] A recent trial by the present research team used a tablet screener to replace the previously used paper screening system and created a registry of smokers, yielding dramatic increases in NRT and Quitline enrollment rates for parents compared to control practices (NRT 38% vs. <1%; Quitline 32% vs. <1% P<.01 for each).[2] Despite such strong and effective results, sustainability of the intervention and impact on parental quit rates have been limited due to the lack of fidelity, systems-level integration, and methods to facilitate connection to external resources. To address the weaknesses of the previous strategy and to best provide care to parents, the proposed project sustainably integrates these changes into child healthcare by developing a disseminable, innovative electronic health record (iEHR)-based approach that supports optimal workflows. Additionally, parents enrolled in the study will be offered assistance by a community health navigator (CHN). This proposed role of a CHN in the child healthcare setting to address household tobacco use has not been studied. This study aims to examine how effective the iEHR + Navigator strategy is compared to usual care control.
**Study Objective(s)**	<u>**Primary:**</u> Parental combusted tobacco quit rate at 1 year, as assessed by validated surveys, biochemically-confirmed at 12-months compared between iEHR + Navigator vs. usual care control.<br><u>**Secondary:**</u><br>1. To compare parents' self-reported adoption of tobacco free behaviors (quit attempts, use of pharmacotherapy, use of Quitlines and institution of smoking and vaping bans in their homes and cars) between the iEHR + Navigator and usual care arms<br>2. To establish the incremental cost per quit of the iEHR + Navigator vs. usual care arms<br>3. To assess the implementation and sustainability of the intervention in the iEHR + Navigator arm (NRT prescription and Quitline referrals as assessed by the parental report around 12-months, the

	screening survey conducted at check-in, and EHR documentation of screening and services delivery fidelity, before and immediately following implementation, 6, 12, 18, 24, 30, and 36 months).
**Test Article(s)**	iEHR + Navigator
**Study Design**	2-arm Randomized Control Trial with a stratified cluster randomization
**Subject Population**<br>**Inclusion and Exclusion Criteria:**	<u>**Inclusion Criteria**</u><br>Parents or legal guardians who smoke, are present at the visit, and whose child is seen by a child healthcare clinician in a participating practice. "Smoker" will be defined as answering "yes" to either of the screening questions: *"Have you smoked a cigarette, even a puff, in the past 7 days?" and "Have you smoked any other tobacco product (cigars like black and mild, hookah), even a puff, in the past 7 days?"* Dual users of e-cigarette or vaping device will also be included. Eligibility will be confirmed using the Eligibility Form.<br><u>**Exclusion Criteria**</u><br>1. Parent/legal guardian does not speak English;<br>2. No telephone; and<br>3. Prior enrollment in the study during a previous visit.
**Number of Subjects**	Total Number of parent subjects: Approximately 800<br>Total number of clinicians and staff: Approximately 40<br>Total Number at CHOP: Approximately 840<br>Total Number of Sites: Minimum 8
**Study Duration**	Each parent participation will last 12 months<br>Each clinician and staff participation in the intervention practices will last 36 months<br>The entire study is expected to last 5 years
**Study Phases**<br>**1. Practice Selection & Randomization**	At least 8 CHOP practice sites will be approached and selected by PeRC network leadership and randomized to: (1) 'iEHR +Navigator' and (2) 'usual care control' arms (4 practices in each arm). In the event that a practice drops out, we will replace it with the most similar practice based on practice size and Medicaid rate from the pool of unassigned and interested practices. In the event the enrollment is slower than projected, we will add new practices that are similar based on practice size and Medicaid rate in each arm.

**2. Parent Screening & Enrollment**	In each practice, study team will review patient medical records and those parents who identified themselves as smokers, will be offered enrollment in the longitudinal cohort. We will continue to enroll parents until approximately 100 parents who use combusted tobacco have been enrolled in the longitudinal cohort in that practice or approximately 400 participants have been enrolled in each arm.
**3. Intervention**	Components of the **iEHR** part of the intervention will include automated EHR-generated NRT prescription, automated enrollment in the free state tobacco Quitline and/or SmokefreeTXT, and a brief motivational message delivered by the clinician (as prompted by the EHR).<br>Additional support of Community Health Navigators (CHNs) will be offered to all parents enrolled in the study. CHNs will provide customized smoking cessation support to household tobacco users through phone follow-up and/or home visits, as requested and as study resources allow.
**4. Follow-Up**	Enrolled parents will be followed-up with a survey at approximately 12-months at their annual check-up or remotely. Parents who self-reported 7-day combusted tobacco quit rates will be offered the cotinine test. In addition to the cotinine test, for parents who report current use of NRT or e-cigarettes or have a cotinine level of ≥10ng/ml, the readings from the carbon monoxide levels test will also be used to confirm self-reported smoking cessation.
**Efficacy Evaluations**	1. At the 12-month follow-up the following will be evaluated: parental 7-day combusted tobacco quit rates and tobacco free behaviors, as assessed by validated surveys; cotinine levels for all parents who self-reported quitting combusted tobacco.<br>2. Delivery and sustainability of interventions will be assessed by 12-month follow-up survey data, pre-visit tobacco screener data, electronic health record data and clinician and staff key informant interviews.<br>3. Incremental cost per quit of the intervention will be assessed by observation and survey of personnel.
**Statistical and Analytic Plan**	The primary outcome will compare the parental combusted tobacco cessation, biochemically-confirmed between the intervention arm (iEHR + Navigator) vs. usual care control. For cotinine confirmed quitting, only self-reported non-smokers who have cotinine values <10 ng/ml, a consensus cutoff level, will be considered quit. In addition to the cotinine test, for parents who report current use of NRT, or e-cigarettes or have a cotinine level of ≥10ng/ml, the readings from carbon monoxide levels test will be also used to confirm self-reported smoking cessation. For CO confirmed quitting, expired-air CO cutoff level of < 5ppm will be

	considered quit.[3] E-cigarette use will be noted and analyzed separately as the literature emerges. In our intention-to-treat analysis, all participants lost to follow-up will be considered smokers. We will also use multiple imputation techniques to impute missing data as a sensitivity analysis, as appropriate.
**Data and Safety Monitoring Plan**	This is a minimal risk clinical trial (as defined in federal regulation at 45 CFR 46.102(i)). As such, we will implement a Data Safety and Monitoring Plan, but not have a formal Data Safety and Monitoring Board.

TABLE 1: SCHEDULE OF STUDY PROCEDURES

YEARS	Year 1				Year 2				Year 3				Year 4				Year 5			
QUARTERS	Q1	Q2	Q3	Q4	Q1	Q2	Q3	Q4	Q1	Q2	Q3	Q4	Q1	Q2	Q3	Q4	Q1	Q2	Q3	Q4
TASKS																				
**Advance Planning, Practice Recruitment**																				
Develop detailed study and recruitment protocols																				
Develop EHR system																				
Pilot and test EHR system																				
Finalize data collection instruments and consent forms																				
Create and test databases																				
Finalize practice training materials																				
IRB submissions (CHOP/MGH)																				
Recruit practices																				
**Data Collection and Intervention Implementation**																				
Launch EHR system and train practice staff																				
Collect EHR documented cessation services data (after impl, 6wk, 12mo, 18mo, 24mo, 30mo, and 36 mo)																				
Navigator hiring and training																				
Hire and Train research assistants for enrollement at the practices																				
Screen and enroll eligible parents																				
Navigators deployed to help parents randomized to iEHR+navigator arm																				
12-month parent exit and phone interviews																				
Cost-effectiveness surveys																				
**Analysis and Reporting**																				
Data monitoring and analysis																				
Process and outcome papers																				
National dissemination activities																				

.

Figure 1: Study Design Schema[1]

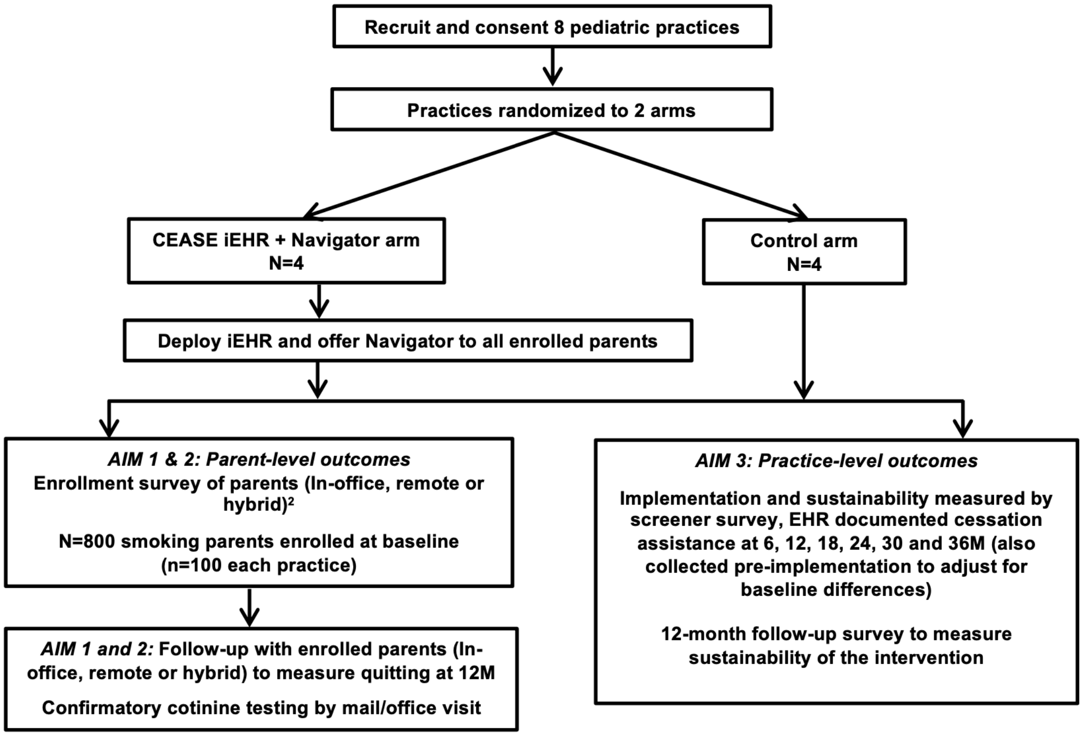

[1]Practices will be randomized to 2 arms, with 4 sites in each arm and ~400 parent subjects per arm (~100 per site). Approximately 800 parent subjects across 8 sites (~100 per site) will be enrolled. Enrolled parents will be followed up at approximately 12 months (could be sooner or later depending on the visit schedule/parent availability).
[2]Parent screening and enrollment procedures may be modified to kiosk/telephone/remote screening and enrollment due to procedures that may be in place at CHOP to protect people from COVID-19 risks.

1 BACKGROUND INFORMATION AND RATIONALE

1.1 Introduction

Helping *parents* quit smoking tobacco is a national priority. When parents and legal guardians (henceforth parents) quit smoking, their life expectancy is increased by an average of >10 years,[4] future tobacco-related poor pregnancy outcomes are eliminated,[5] their children have much lower odds of becoming smokers,[6–10] and their children may no longer be exposed to high levels of tobacco smoke, decreasing the odds of contracting diseases caused by tobacco smoke exposure (TSE) and resulting in fewer missed school days.[11] Helping parents quit smoking also improves the financial resources of families,[12] decreases the risk of developmental delays,[13,14] and lowers risk of house fires.[15] Parents who smoke are often medically underserved, visiting their child's clinician more often than they see their own, if they even have one.[16] In addition, dual use of electronic and traditional cigarettes has been increasing, putting children at increased risk of unhealthy aerosol exposures.[17,18] Despite this extraordinary opportunity for intervention, child healthcare settings deliver effective tobacco dependence treatment to parents < 2% of the time.[1] Routinely delivered tobacco treatment to parents in child healthcare settings would benefit the overall health of the nation, especially vulnerable populations who suffer the majority of the 480,000 annual US preventable deaths.[14]

The effectiveness of smoking cessation strategies is well established, but these methods are underutilized. The September 2015 "A" recommendation from the US Preventive Services Task Force indicates high certainty of benefit resulting from clinicians asking all adults about smoking, prescribing FDA approved cessation medications for those who smoke, and offering appropriate behavioral interventions.[19] Background quit rates for the US population are up to 5% per year.[20] Cessation strategies, offered in combination, greatly improve the likelihood of success. Physician counseling alone yields a quit rate of 19.9% [13.7-26.2], the Quitline alone a cessation rate of 12.7% [11.3-14.2], and using combination nicotine replacement therapy (NRT) patch and *ad lib* NRT gum medications a cessation rate of 36.5% [26.6-45.3].[21] Moreover, the combination of these therapies, as will be used in the proposed intervention, is more effective than individual components alone.[21] In addition, a meta-analysis recently demonstrated the effectiveness of parental smoking cessation initiated in the pediatric context.[22] Yet household-centered, systematic tobacco control interventions to support parents in quitting are not being used in most child healthcare settings.

The Clinical Effort Against Secondhand Smoke (CEASE) intervention effectively increases delivery of tobacco cessation interventions for parents, but innovations to sustain the intervention are needed. The CEASE intervention has been successfully developed and implemented to train child healthcare clinicians to address household smoking behavior, and promote smoke-free and e-cigarette free home and car rules in a

routine and effective manner.[18,23–26] Child healthcare offices are trained to change their office system to address parental tobacco use, connect smokers to free resources, and prescribe medications for cessation.[1,27,28] The training is conducted through online American Academy of Pediatrics (AAP) continuing education modules, in-person training, phone calls, videos, and reference materials, based on the practice needs.[25,29,30] Our most recent trial R01CA127127 used a tablet screener to replace the previously used paper screening system and created a registry of smokers, yielding dramatic increases in NRT and Quitline enrollment rates for parents compared to control practices (NRT 38% vs. <1%; Quitline 32% vs. <1% P<.01 for each).[27,31] Despite such strong and effective results, sustainability and impact on parental quit rates have been limited due to the lack of fidelity, systems-level integration, and methods to facilitate connection to external resources. To address the weaknesses of the previous strategy and to best provide care to parents, the proposed project sustainably integrates these changes into child healthcare by developing a disseminable, innovative electronic health record (iEHR)-based approach that supports optimal workflows.

1.2 Name and Description of Investigational Product or Intervention

The iEHR + Navigator intervention consists of an innovative electronic platform for smoking screening and automated smoking cessation support to parents who smoke. iEHR will include automated EHR-generated NRT prescription, automated enrollment in the free Quitline, and a brief motivational message delivered by the clinician (as prompted by the EHR) when a parental smoker is identified. The enrolled parents will also be offered support from CHNs, who will work with offices to provide customized cessation support to household tobacco users, ensuring access to medications and services.

1.3 Relevant Literature and Data

Evidence for CEASE: Using a paper-based system, the MGH team's previous parental tobacco control trial (R01-CA127127) demonstrated that effective parental tobacco control intervention (CEASE) can be implemented in child healthcare practices by using existing systems of care (see A3).[1] We subsequently tested the potential benefit of electronic screening with CEASE using an electronic tablet (adaptable to other formats (e.g., kiosk, smartphone)) to identify parental smokers and delivery of treatment by the child healthcare clinician (see Figure 1). The intervention was effectively implemented in five child healthcare practices in five states (OH, IN, TN, VA, NC; N=1002 parents) and compared to five control practices in the same states (N=936 parents). 70% of parents in intervention practices reported being screened for tobacco use vs. 22% in the control practices. Practices' delivery of parental tobacco control assistance (prescription of medication, 34% vs. 0%, referral to the state Quitline, 25% vs. 0%) was markedly higher in intervention practices post-implementation (P<.0001).[31] At two years post-implementation, 1007 parents in intervention practices and 943 in control completed the exit interview. Practices' delivery of parental tobacco control assistance (prescription of medication, 10% vs. 0%, referral to the

state Quitline, 7% vs. 0%) was still markedly higher in the intervention (P<.0001) but had dropped from immediate post-implementation levels.[32] Overall, the team demonstrated a 3.7% net reduction in population smoking rate relative to control practices after two years.[32] Despite such strong results, intervention practices did not fully adopt and sustain tobacco control service delivery. Critical strengths of the proposed trial address these weaknesses with automated household tobacco use screening that integrates into the EHR, delivers tobacco dependence treatments through the iEHR intervention, and tests the provision of additional support from a CHN.

EHR-based tobacco cessation tools are feasible, acceptable, usable, and impact clinical care. In a prospective study of pediatric clinicians and parents at one practice, the CHOP team developed and evaluated the feasibility, acceptability, usability, and clinical impact of a tool within the EHR to help pediatric primary care clinicians provide smoking cessation treatment to parents who smoke.[33] Clinicians received training in use of the EHR tool. The tool prompted clinicians to ask about tobacco smoke exposure (TSE) at all visits, to print a prescription for NRT gum and patch, and to refer to a free, external cessation program. Pediatric clinicians used the screening tool at 2286 (76%) of 3023 visits. Parent smokers were identified at 308 visits; 55% of smokers were interested in and offered treatment. 24 (80%) of 30 eligible pediatric clinicians used the tool. Indicating that clinicians uniformly found the tool helpful, 94% were satisfied with the tool, and the average System Usability Scale score[34] was 83/100 (good to excellent range). Of a sample of 100 parents who received treatment, 69 parents were reached and surveyed, with 44 (64%) receiving NRT prescription. In a randomized controlled trial involving 484 parent smokers at the same urban site, the CHOP team subsequently demonstrated that an electronic referral to the Quitline, enabled through the EHR, resulted in a significant 8.3 percentage point greater enrollment in the Quitline compared to a paper-based referral, a 5-fold increase.[35] The current R01 incorporates the successful electronic screening and referral strategy above in the iEHR to support automated cessation workflows.

Navigators arranged through child healthcare offices are well-received and improve outcomes. Drs. Bryant-Stephens, Collins, and Winickoff completed a care coordination study at CHOP. Community health workers served as tobacco control navigators. Parents were randomized either to receive personalized phone counseling from a navigator to promote cessation and SHS protections or to an attention control intervention.[36] The navigator conducted phone counseling over two weeks and reinforced clinic-level messaging. The navigator also provided support, education, and cognitive-behavioral skills training to promote TSE reduction and parent cessation. In the overall sample (N=327), there was a marked difference in the homes with smoking restrictions from baseline to 3-month end-of-treatment (96% Intervention vs 81% Control; P<.001). Significantly more participants in the navigator condition reported 7-day quit rate (28% Intervention vs. 8%

Control; p<.001). The proposed study will adapt this feasible, acceptable, and efficacious model to deliver CHN assistance for tobacco dependence, helping to ensure smoking parents and household members get what they need to quit.[37] The study will test the effectiveness of CHN support added to the iEHR intervention.

1.4 **Compliance Statement**

This study will be conducted in full accordance all applicable The Children's Hospital of Philadelphia Research Policies and Procedures and all applicable federal and state laws and regulations including 45 CFR 46 and the Good Clinical Practice: Consolidated Guideline approved by the International Conference on Harmonisation (ICH). All episodes of noncompliance will be documented.

The investigators will perform the study in accordance with this protocol, will obtain consent and assent, and will report unanticipated problems involving risks to subjects or others in accordance with The Children's Hospital of Philadelphia IRB Policies and Procedures and all federal requirements. Collection, recording, and reporting of data will ensure the privacy, health, and welfare of research subjects during and after the study.

2 STUDY OBJECTIVES

To compare the effectiveness of the iEHR + Navigator versus usual care control, we propose a 2-arm RCT with a stratified cluster randomization of child primary care practices with 4 practices each randomized to iEHR + Navigator or usual care control arms.

2.1 **Primary Objective**

Parental combusted tobacco quit rate at 1 year, as assessed by validated surveys, biochemically-confirmed at 12-months compared between iEHR + Navigator vs. usual care control.

Hypothesis. Parental 7-day combusted tobacco quit rates, as assessed by validated surveys, biochemically-confirmed at 12-months, will be greater for iEHR + Navigator vs. usual care control (outcome for which study is powered).

2.2 **Secondary Objectives.**

The secondary objectives are to:

1. To compare parents' self-reported adoption of tobacco free behaviors (quit attempts, use of pharmacotherapy, use of Quitlines and institution of smoking and vaping bans in their homes and cars) between the iEHR + Navigator & usual care arms
2. To establish the incremental cost per quit of the iEHR + Navigator vs. usual care arms
3. To assess the implementation and sustainability of the intervention in the iEHR + Navigator arm (NRT prescription and Quitline referrals as assessed by the parental report around 12-months, the screening survey conducted at check-in, and EHR

documentation of screening and services delivery fidelity, before and immediately following implementation, 6, 12, 18, 24, 30, and 36 months).

3 INVESTIGATIONAL PLAN

3.1 General Schema of Study Design

This is a stratified cluster randomized controlled trial of minimum 8 child primary care practices with minimum 4 practices each randomized to iEHR + Navigator or usual care control arms:

Figure 1: Study Design Schema[1]

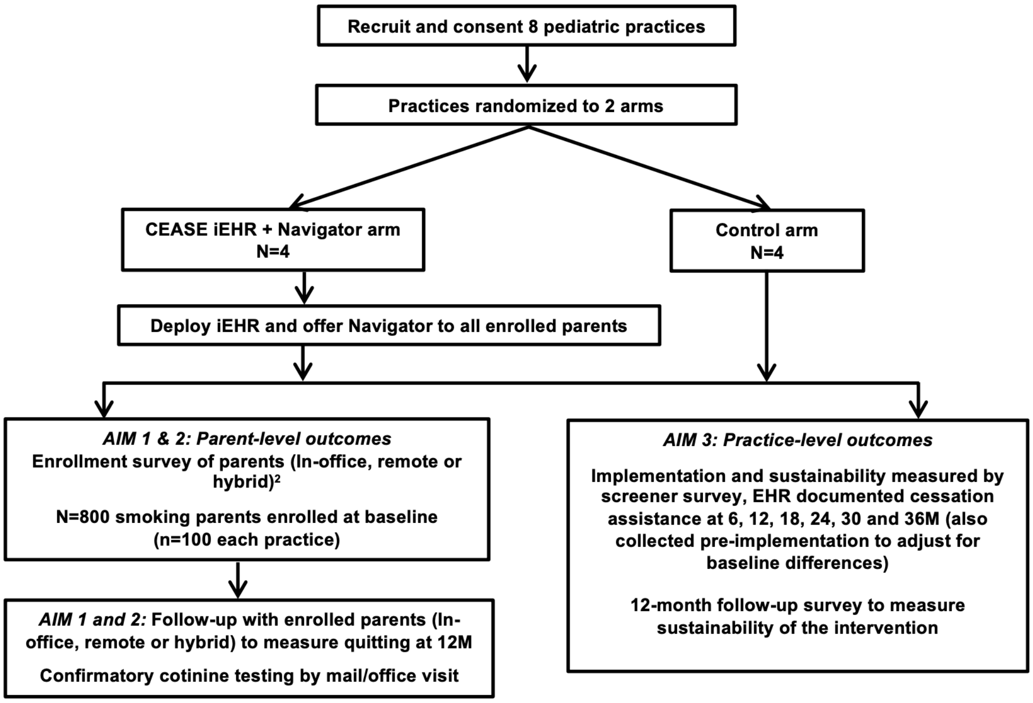

[1]Practices will be randomized to 2 arms, with 4 sites in each arm and ~400 parent subjects per arm (~100 per site). Approximately 800 parent subjects across 8 sites (~100 per site) will be enrolled. Enrolled parents will be followed up at approximately 12 months (could be sooner or later depending on the visit schedule/parent availability).

[2]Parent screening and enrollment procedures may be modified to kiosk/telephone/remote screening and enrollment due to procedures that may be in place at CHOP to protect people from COVID-19 risks.

3.1.2 Parent Screening & Enrollment

Control and intervention practices will be trained in integrating Research Assistants (RAs) who will be hired, trained, and paid by the research study for data collection. A notice may be posted at the practice entrance/registration window/waiting room of participating practices, informing parents accompanying their children to office visits that the practice is participating in the research study (posting details may be modified based on practice layout and preferences). We will deploy several in-person (at CHOP pediatric primary care) and remote (via e-mail, phone and/or text) strategies for screening and enrolling smoking parents to maximize recruitment efforts.

The study team will review patient medical records and those parents who identified themselves as smokers, will be approached by RAs and offered enrollment in the longitudinal cohort. We will continue to enroll parents until approximately 100 parents have been enrolled in the longitudinal cohort in that practice. Parent screening and enrollment procedures may be modified to kiosk/telephone/remote screening and enrollment due to procedures that may be in place at CHOP to protect people from COVID-19 risks.

The detailed description of the screening and enrollment procedures is under *section 4.1 Screening & Enrollment.*

3.1.3 Intervention

The intervention consists of an innovative EHR platform, iEHR, which will be used to screen families for tobacco use and refer smoking parents to smoking cessation resources and treatment. Components of the iEHR will include automated EHR-generated NRT prescription, automated enrollment in the free Quitline and/or SmokefreeTXT program, and a brief motivational message delivered by the clinician (as prompted by the EHR). As an added service, it may also include a referral to the company called BrightMedical that will facilitate the delivery of the NRT medication to interested families. Community Health Navigator support will be offered to all smokers enrolled in the intervention arm. CHNs will provide customized smoking cessation support to household tobacco users through phone follow-up and/or home visits, depending on the needs of each family and as study resources allow.
See a detailed description of the iEHR + Navigator intervention and its components under *section 4.2.1.*

3.1.4 Follow-Up

Enrolled parents will be followed-up with a survey after approximately 12-months. Those parents who do not return at annual check-up will be contacted remotely (e.g., phone, text, HIPPA-compliant video system, email, portal (myCHOP) messaging). Parents who self-report 7-day abstinence at the 12-month surveys (in-person or by phone/text) will be asked to submit their saliva for cotinine testing. In addition to the cotinine test, for parents who report current use of NRT or e-cigarettes or have a cotinine level of ≥10ng/ml, the readings from the carbon monoxide levels test will also be used to confirm self-reported smoking cessation.

See a detailed description of the follow-up procedures under *section 4.3 Follow-Up.*

3.2 Practice Selection, Randomization and Blinding

At least eight CHOP practice sites will be approached and selected by PeRC network leadership.

All practices are eligible to participate. We will preferentially enroll practices with the following criteria:

1. A minimum 10% Medicaid insurance rates for children
2. An average patient flow of 40 families per day
3. A willingness to have a Research Assistant in the office setting

Eligible practices will be randomized to: (1) 'iEHR + Navigator' and (2) 'usual care control' arms (4 practices in each arm). Practices will be matched based on practice size (based on the number of children seen in a typical year (this will account for COVID-19)) and the Medicaid rate at the practice.

In this 2-arm cluster randomization of practices to 'iEHR +Navigator' and 'usual care control' arms (4 each), we will recruit four matched pairs of practices through the CHOP network. Practices will be matched with respect to size and Medicaid rate. Within each of these pairs of practices, we will assign one to the intervention and the other to control, using computer generated random allocation. When the four assignments are complete, a fifth computer-generated random number will assign either "A" or "B" to the intervention. The statisticians will be blinded as to which of "A" and "B" is the intervention until the analyses are completed. In the event that a practice drops out, we will replace it with the most similar practice based on practice size and Medicaid rate from the pool of unassigned and interested practices. In the event the enrollment is slower than projected, we will add additional practices from the CHOP network, with practices matched in pairs based on practice size and Medicaid rate, from the pool of unassigned and interested practices. We will randomly allocate these practices in pairs to the control and intervention arms, as needed and in decreasing order of projected enrollment rate.

A practice leader will be identified in each practice. This person will be the study team's contact person at the practice.

At the end of data collection, all control practices will be offered the iEHR intervention.

3.3 Study Duration, Enrollment and Number of Sites

3.3.1 Duration of Study Participation

Each parent participation will last 12 months.

Each clinician and staff participation in the intervention practices will last 36 months. Clinician and staff participation and study activities are described in detail under section *4.3.3 Practice Clinicians and Staff Qualitative Interviews.*

Practice data will be collected for approximately 36 months post implementation.

The sections below will focus on the parents' participants.

3.3.2 Total Number of Study Sites/Total Number of Subjects Projected

The study will be conducted at a minimum of 8 primary care sites at CHOP with approximately 40 clinicians participating across these 8 sites, and proportionally more clinicians if other sites are added.

Our goal is to stop recruitment when 400 parent subjects are enrolled per arm in the longitudinal cohort (800 across both arms). The study has been designed to achieve this goal by recruiting 100 parents from each of four sites per arm, and for this situation we will have 80% power for the primary outcome (iEHR + Navigator vs. usual care control), for a clinically meaningful difference in smoking cessation rates. If some practices accrue subjects at a rate which makes it unlikely that these practices can achieve the recruitment goal of 100 subjects, then we will estimate the likely shortfall, and increase the recruitment goals in faster-accruing practices in order to attempt to preserve statistical power. We may also add additional practices to maximize the likelihood of meeting the recruitment goal of at least 400 parents enrolled in each arm. If we add practices, we will do so in pairs, matched by practice size and Medicaid rate, and randomly assigned to the control and intervention groups. If the study must be terminated before the recruitment goal, this termination will be made exclusively for reasons (such as time and budget) which are unrelated to study outcomes.

In the intervention practices, a minimum of one clinician and one key staff will complete an in-depth qualitative interviews 6-weeks following implementation and after 12, and 24 months.

3.4 Study Population

3.4.1 Inclusion Criteria

We will enroll parents or legal guardians who smoke, are present at the visit, and whose child is seen by a clinician in the study practice. "Smoker" will be defined as answering "yes" to either of the screening questions: *"Have you smoked a cigarette, even a puff, in the past 7 days?" and "Have you smoked any other tobacco product (cigars like black and mild, hookah), even a puff, in the past 7 days?"* on the iEHR pre-visit screening

questionnaire (Appendix 5 for intervention practices and Appendix 5A for control practices). The eligibility criteria will be confirmed using Eligibility Form (Appendix 10) prior to consent and enrollment. Parents will also be screened for JUUL/E-cig use. Each eligible parent who indicates cigarette and/or other tobacco product use in the past 7 days will be allowed to enroll in the study, regardless of whether or not the parent uses any type of e-cigarette or vaping device.

3.4.2 Exclusion Criteria

1) Parent/legal guardian does not speak English; 2) No telephone; and 3) Prior enrollment in the study during a previous visit.

Subjects that do not meet all of the enrollment criteria may not be enrolled.

Over the course of the study, parents who screened positive for smoking on the iEHR pre-visit screening questionnaire, are prompted to complete it every 3 months if they have a visit. Additionally, parents with multiple children, are prompted to complete the iEHR pre-visit screening questionnaire for each child. As a result, parents may show up as eligible for the study in the EHR multiple times despite being already enrolled in the study. We have implemented several safeguard mechanisms to avoid duplicate enrollments of such parents (checking the reports of already enrolled parents, asking parents whether they have already been enrolled in the study). Nevertheless, it is difficult to completely avoid duplicate enrollments, especially when a parent chooses not to disclose that they are already taking part in the study. The study team will monitor the REDCap projects regularly to identify any incidents of duplicate enrollment. If a duplicate enrollment is identified, the study team will notify the parent that they can be enrolled in the study only once and their information will be kept only under the original enrollment. The study team and the investigator will keep track of the duplicate enrollments to ensure validity of the data, and only use the data from the initial enrollment. This study is minimal risk, and the second enrollment does not disqualify a parent from being kept in the study under the original enrollment.

Any other violations of these exclusion criteria must be reported in accordance with IRB Policies and Procedures.

4 STUDY PROCEDURES

4.1 Screening & Enrollment

The protocol for the recruitment, consent, and retention of participants will be identical in all practices. As part of the clinical workflow, all parents in control and intervention practices will be asked about their individual smoking status on the iEHR pre-visit screening questionnaire (Appendix 5 for intervention practices and Appendix 5A for control practices). We will deploy several strategies to maximize screening and enrollment efforts.

The study team/CHOP Clinical Reporting Unit (CRU) and/or Research Enhancement Core (REC) will generate rosters of potentially eligible parents for the upcoming visit. Rosters will include all upcoming visits at study practices of parents who answered, “yes” to either of the screening questions: *“Have you smoked a cigarette, even a puff, in the past 7 days?” and “Have you smoked any other tobacco product (cigars like black and mild, hookah), even a puff, in the past 7 days?”* on the iEHR pre-visit screening questionnaire. The study team will offer enrollment into the study to these potentially eligible parents in-person at the primary care site, via the phone or e-mail.

Additionally, at participating CHOP primary care sites, study team will also review the medical records to identify parents who self-reported as smokers (answered “yes” to either of the screening questions: *“Have you smoked a cigarette, even a puff, in the past 7 days?” and “Have you smoked any other tobacco product (cigars like black and mild, hookah), even a puff, in the past 7 days?”* on the iEHR pre-visit screening questionnaire. (Appendix 5 for intervention practices and Appendix 5A for control practices) to approach them and offer enrollment in the study longitudinal cohort. If a parent is not interested in participating in the study, the research team will document this information in REDCap (child’s name, MRN, parent’s relationship to the child, practice name and date of the visit).

Potentially eligible families who are missed in the CHOP primary care sites will also be reached via recruitment emails, text messages and calls facilitated by the study team and/or CHOP Research Enhancement Core (REC). All outreach methods (e-mail, texts, calls) will include information about how parents can opt out from being contacted about this study in the future.

Smoking parents will by recruited in one of these ways:

1. **In-office enrollment**: At participating CHOP primary care sites, study team will approach eligible parents i.e. those who self-reported as smokers (answered “yes” to either of the screening questions: *“Have you smoked a cigarette, even a puff, in the past 7 days?” and “Have you smoked any other tobacco product (cigars like black and mild, hookah), even a puff, in the past 7 days?”* on the iEHR pre-visit screening questionnaire (Appendix 5 for intervention practices and Appendix 5A for control practices) to approach them and offer enrollment in the study longitudinal cohort. Study team will confirm eligibility criteria using Eligibility Form (Appendix 10) prior to consent and enrollment. If a parent agrees, they will be consented using Informed Consent of Parent (Appendix 3b and 3bE (for intervention group) and 3 and 3E (for control group)). After consent, parents in the intervention arm will also be offered the additional support of CHOP CHNs (Attachment 11) (see section 4. 2.2. Community Health Navigators for more detail). Parent Enrollment Survey (Appendix 4) will be then administered by the RA in both intervention and control

practices. The Parent Enrollment Survey (Appendix 4) includes additional questions assessing parental vaping status, stage of readiness to change, demographic information, tobacco related attitudes and behaviors and parents' contact information (~ 15 minutes).

In-office enrollment for parents that don't complete the iEHR pre-visit screening questionnaire: At participating CHOP primary care practices, screening questionnaires are assigned to a patient record as opposed to each parent and that sometimes results only in one parent being screened for smoking as opposed to each parent. The study team will fill these gaps by offering enrollment into the study for every smoking parent or legal guardian present at the visit.

2. **Hybrid enrollment from in-office to remote:** If during the office visit, the parent who was screened positive for smoking and eligible for the study (answered "yes" to either of the screening questions: *"Have you smoked a cigarette, even a puff, in the past 7 days?" and "Have you smoked any other tobacco product (cigars like black and mild, hookah), even a puff, in the past 7 days?"* on the iEHR pre-visit screening questionnaire (Appendix 5 for intervention practices and Appendix 5A for control practices) doesn't have time to complete enrollment or prefers to complete enrollment at home, the study team will ask the parent if he/she is interested in remote enrollment and will ask to collect their contact information for remote enrollment.

Hybrid enrollment if consented in office: If the eligible participant's consent was completed during the visit but the participant doesn't have time to complete the enrollment survey, we will follow up with them remotely to complete the survey or complete the survey during a future visit to the participating office.

All contact information will be stored and shared in a secure HIPAA compliant way (i.e., using CHOP OneDrive). The study team will then attempt to follow up with the parent(s) and conduct enrollment remotely.

3. **Remote enrollment:** We will also deploy additional recruitment approaches, including calling, emailing and texting eligible participants (those who answered "yes" to either of the screening questions: *"Have you smoked a cigarette, even a puff, in the past 7 days?" and "Have you smoked any other tobacco product (cigars like black and mild, hookah), even a puff, in the past 7 days?)"* on the iEHR pre-visit screening questionnaire at the study practices. The roster of eligible participants will

be generated by the study team and/or CHOP REC. Parents that were already enrolled in the study will be excluded.

For outreach calls, the study team will briefly explain the purpose of the call and introduce the study prior to consent. If the parent agrees to participate, the consent and survey will be sent to them electronically via text or e-mail. During the phone outreach, parents will not be texted or e-mailed the study materials without verbally agreeing to it during the call. All outreach emails and texts will contain the REDCap link inviting them to consider participation in the study. Parents who choose to participate will be screened for eligibility via the REDCap survey (Eligibility Form - Appendix 10) and they will provide informed consent/HIPAA authorization electronically (Appendix 3bE (for intervention group) and 3E (for control group). After they electronically sign the consent, parents in the intervention arm will also be offered the additional support of CHOP CHNs (Attachment 11) (see section 4. 2.2. Community Health Navigators for more detail). Then parents in both intervention and control groups can complete the enrollment form (Appendix 4) either at home themselves or while having a research assistant on the call. If the parent picks up the call but it's not a good a time for them to complete the enrollment, the RA may offer them an option to receive follow up with the RA via text to set up the best time for the enrollment call. The research presents minimal risk to participants and involves no procedures for which written consent is normally required outside of the research context.

If the parent has a working e-mail address on record, we may also attempt to outreach to them via e-mail (e-mail template: Appendix 12 for intervention and Appendix 13 for control). E-mail letters may be customized for each practice based on the input from practice physicians and staff. E-mail letters will include an option to opt-out from further communication and will notify the parents that if they do not enroll into the study or opt out, within 2 weeks we may follow up with them via phone call or text. Follow up texts (with up to 3 reminders if the parent doesn't opt out) will include a brief information about RAs call back number to learn more about the study. Parents may opt out at any time.

Using the workflows outlined above, we will continue to enroll parents until approximately 400 parents have been enrolled in each arm. If some practices accrue subjects at a rate which makes it unlikely that these practices can achieve the recruitment goal of 100 subjects, then we will estimate the likely shortfall, and increase the recruitment goals in faster-accruing practices in order to attempt to preserve statistical power. We may also add additional practices in one or both arms to meet the recruitment goal of 400 parents enrolled in each arm. A notice to parents

may be posted where all can see it when entering/registering for their pediatric visit. The notice may announce that the practice is participating in a research study and that parents/guardians may be approached by a study RA during their office visit. Parent screening and enrollment procedures may be modified to kiosk/telephone/remote screening and enrollment due to procedures that may be in place at CHOP to protect people from COVID-19 risks.

4.2 **Intervention**

4.2.1 iEHR

The iEHR intervention consists of an innovative electronic platform for smoking screening and automated smoking cessation support for parents who smoke. Elements of iEHR will be tailored to fit practices' workflow, staffing, resources, and physical configuration. The intervention involves training all office staff to utilize the iEHR system to assist all parents who smoke to support sustainable workflows and reduce the clinician burden. Components of the iEHR will include:

(1) Identification of smokers and self-assessment of quitting preferences: Screening for parent smoking in the intervention arm will be expanded upon using the iEHR pre-visit screening questionnaire (Appendix 5). The final wording and look of the questionnaire may be updated when the technical team will start integrating it in the EHR. Parents will complete the questionnaire prior to their visit with the clinician, either via MyCHOP prior to the office visit or on a tablet or their own device once they arrive to the office. All parents will receive this pre-visit screening questionnaire. We will determine the appropriate frequency of the screener, so that it's not burdensome to the parents and does not disrupt practice workflow. This workflow, which is already occurring and accepted widely, enhances the role of front desk staff to ensure that clinical information is available in the chart prior to the visit with the clinician.

(2) Counseling: Upon review of the pre-visit screener results, the iEHR will automatically document a parent's intention to quit and services requested to help them quit and suggest motivational messages to increase parents' acceptance of services.

(3) Automatically generated prescription for NRT/NRT delivery: the iEHR will automatically generate a prescription for NRT for parents who smoke (unless they opt out of receiving the prescription), including for those cutting down to quit as recommended in latest guidelines.[38] Prescriptions for two forms of NRT (patch and gum) will be printed. Based on information available in the EHR for the parent who completed the pre-visit screening questionnaire, all identifying fields for the prescription will be populated. Prescriptions will be either handed over to the parents or faxed/electronically transmitted to their pharmacy or NRT will be mailed/delivered to them. We may use the services of the company called, BrightMedical, to facilitate the delivery of the NRT medication to families.

(4) Automated Quitline and SmokefreeTXT enrollment:

In the CEASE pre-visit screening questionnaire (Appendix 5), smoking parents will be offered automatic enrollment in the state's free tobacco Quitline and the SmokefreeTXT program, a text message program offered by the National Cancer Institute.

4.2.2 Community Health Navigators (CHNs)

Table 2. Community Health Navigator role

CHN session	**Ask**[25]<br>(Assessment)	**Assist**<br>(Counseling)	(Care coordination)	**Refer**<br>(Resources)
1	Identify all household tobacco users<br>• Readiness to quit<br>• Smoke-free home & car rules<br>• Previous quit attempts<br>• NRT insurance coverage<br>• Identify pharmacy	Enhance intervention content:<br>• Quit smoking tools & techniques for all tobacco users<br>• Thirdhand smoke<br>• Set quit date	• Ensure connection to Quitline<br>• Ensure NRT access at local pharmacy<br>• Communicate with child's clinician & if possible, parents' clinician about household smoking status using iEHR	Follow-up to ensure resource awareness & receipt of:<br>• NRT scripts<br>• Quitline<br>• SmokefreeTXT
2	• Reassess motivation and progress<br>• NRT script filled<br>• Connected with Quitline/ local cessation resources<br>• Assess smoke-free zones	Reinforce intervention content and all of the above	• All of the above	• Develop strategies to overcome barriers to service receipt

Parents in the intervention arm will also be offered the additional support of CHOP CHNs (Appendix 11). CHNs are employees of CHOP Community Asthma Prevention Program (CAAP) led by the co-PI of this study Dr. Tyra Bryant-Stephens. CHNs are listed on the CHOP study team and when additional CHOP CHNs are hired for this study, they will be added to the study team. CHNs will work with offices to provide customized smoking cessation support to household tobacco users through phone follow-up, ensuring access to medications and services, and/or home visits, depending on the needs of each family and as study resources allow (Table 2). Home visits could be virtual if there are in-person visit restrictions due to COVID-19.

The CHN will contact the smoker within approximately one week following enrollment to set up the first telephone, video, or in-home session, if they are interested. At the first session, the navigator will discuss various types of services available and work with the smoker to determine their best cessation plan. The CHN will ask smokers to set aside up to 1 hour for the session. The second session will be scheduled for approximately 2-4 weeks later, depending on need and availability. After the first session, the CHN will make phone calls or texts to review progress of cessation activities, offer support, and connection to resources. After the second session, these calls will occur as needed for 1 year. The CHN will document any contact made in a standard EHR patient outreach encounter that will be sent to the child's clinician. Using the EHR, clinicians will also be able to message the CHN.

Depending on parent preference, a session could include a telephone or video call with the CHN or home visit, if requested. Home visits could be virtual if there are in-person visit restrictions due to COVID-19.

4.3 **Follow-up**

4.3.1 Parent Follow-up

For smoking parents in the longitudinal cohort (See Figure 1), 12-month follow-up in-person/remote survey (Appendix 6), and biochemical validation of self-reported 7-day abstinence will be obtained using proven protocols to collect the samples in-person at the practice or by using remote survey and mail-in cotinine swabs for parents not present at the practice.

- Cotinine levels will be measured for all parents who reported quitting smoking
- In addition to the cotinine test, parents who report current use of NRT or e-cigarettes or have cotinine levels ≥10 ng/ml will have the option to get carbon monoxide levels tested to confirm combusted tobacco abstinence.
    - For in-office follow ups (see below for more details), to prevent missing parents who might be eligible for carbon monoxide test, we may ask parents who report current use of NRT or e-cigarettes to get their carbon monoxide levels tested in-office without knowing their cotinine levels yet. We will use their carbon monoxide test readings only if their cotinine levels are ≥10 ng/ml (we will get the cotinine results from the lab).

The enrolled parent will be eligible for the 12-month follow-up, about three-weeks before their 12-month study enrollment date. We will deploy several strategies to maximize follow-up efforts.

We will generate rosters of enrolled parents due for their 12-month follow up, who have an upcoming visit scheduled during the follow-up window. These reports will be regularly updated to reflect newly scheduled appointments.

Enrolled parents eligible for follow-up will complete the follow-up assessment in one of the following three ways:

1. **Follow-up via text/email/portal message(myCHOP):** Parents who consented to texting on the baseline Parent Enrollment Survey (Appendix 4) may be texted a follow up survey link. Participants may receive several text reminders to remind the parent to complete the follow up survey. Parents may also be sent the follow up survey link by email or through portal messaging (myCHOP).

   If the parent completes the survey via text, email, or myCHOP,and is eligible for the cotinine swabs and CO test, the last page of the survey will notify them that they

should expect to receive a pre-paid mail envelope with the swab collection kit and a CO test monitor and that we may follow up via phone calls/text/email/myCHOP with additional instructions. If the parent has not mailed their salivary cotinine swab kit or not uploaded their CO readings (if eligible), then, we may approach them at the practice in-person at any upcoming appointment to complete the pending assessments.

If the parent hasn't completed the follow-up survey in-person or remotely and the 12-month follow-up date has passed and there is no scheduled appointment in the next couple of months, we may continue to try to follow-up with the participant remotely.

2. **In-office follow-up:** At the participating CHOP primary care sites, study team may approach eligible enrolled parents whose visit falls during the follow up period window. To maximize opportunities for follow up, if the parent has a scheduled visit at any CHOP locations (e.g., CHOP specialty care), the study team may also approach parents that haven't completed follow up procedures at that CHOP location. The study team will first ask for parent's permission to conduct follow up at that CHOP location. Only if the parent feels comfortable, the study team will proceed with follow up.

   The study team will complete the follow-up survey and if the parent is eligible will offer the cotinine swabs and carbon monoxide breath tests for in-person completion. If not mailed to the lab immediately, the cotinine tests may be stored in a secure location at the corresponding primary care site or at the study team's CHOP office (Roberts) until they are mailed to the lab by the study team.

3. **Phone follow-up:** The study team may call parents to complete their follow-up survey remotely. During the call, the study team member will briefly remind participants of their enrollment, and the purpose of the call before completing the follow-up survey. If the study team is not able to get in touch with the subject via phone, they may send them one or more postcards to remind them of their study participation and encourage them to call/text back to complete the follow-up survey and get their incentive. The study team may also look for updated contact information of the parent in the child's medical record.

   If the parent completes the survey remotely via phone and is eligible for the cotinine swabs and CO test, the last page of the survey and the study team member will notify them that they should expect to receive a pre-paid mail envelope with the swab collection kit and, if eligible, a CO test monitor. They will also be told that they may receive a follow up phone calls/text/email/portal message with additional instructions after the kits have been mailed. If the parent has not mailed their salivary cotinine swab kit or not uploaded their CO readings (if eligible), then, we may approach them at CHOP in-person at any upcoming appointment to complete the pending assessments.

4.3.2 Ongoing EHR Documentation & Evaluation

The EHR documentation of screening and services delivery fidelity, will be ongoing throughout the study before and immediately following implementation, 6, 12, 18, 24, 30, and 36 months. This data is not available from the longitudinal cohort as it requires an assessment of care provided at the practice-level. Specifically, we want to know what services are being delivered at the follow-up time period in the practice overall (as opposed to the limited subset of the practice that it is in the longitudinal cohort).

4.3.3 Practice Clinicians and Staff Qualitative Interviews

In-depth qualitative interviews will be conducted with clinicians and key staff from the intervention practices 6-weeks following implementation and at 12, and 24 months. Clinicians and clinical staff may be recruited for qualitative interviews via e-mail, referral from a colleague, flyer or through a personal solicitation. Prior to the interviews, participants will be offered an information sheet (Appendix 2). The interview guide (Appendix 7) will be based on questions from RE-AIM tools and the CFIR (Consolidated Framework for Implementation Research) interview guide. The interview will be conducted to collect data on the factors that influence intervention implementation. We will also use data from the interviews to improve implementation and sustainability through tailoring the intervention.

The interview process will follow a standard procedure, which is outlined as follows:
Intervention Practice Respondents:

- As a condition of participation in the study, clinicians and staff who will complete the qualitative interviews will complete consent forms (Appendix 8).
- Interviews will be scheduled with selected clinicians and staff at intervention practices. These selected clinicians and staff we expect to include the practice champion and the administrative staff member most involved in the implementation of the intervention; other interviews may be scheduled, if the practice has capacity for additional interviews.

The interviews will be conducted in person, by phone, or HIPPA-compliant video conferencing. The qualitative researcher conducting interviews will begin each interview by asking permission to record, outlining the purpose of the interview, describing the rights of respondents to stop the interview or recording at any time, and giving a brief explanation of how interview data will be used. Interviews will only be recorded with permission.
Recordings will be uploaded to the transcription service provider (ADA Transcriptions). ADA Transcriptions is a HIPAA-compliant, full-service transcription company specializing in medical and academic research. Additionally,

CHOP has a Master Business Associate agreement with the ADA Transcription. The audio-recordings will be uploaded and temporarily stored on ADA Transcriptions online platform that is encrypted and HIPAA compliant. ADA Transcriptions will de-identify and complete the transcriptions and provide each audio-recording and transcription a filename and password-protect it. The password protected transcriptions will then be securely transferred to the designated research team listed on the online platform system. Only designated CHOP and MGH study team staff will be given access to transcriptions. The interview data will be used anonymously in conference presentations, articles, and reports. As part of the rapid-response quality improvement, data will also be used to improve the intervention and address factors that influence implementation of the intervention.

4.4 Subject Completion/Withdrawal

Parents may withdraw from the study at any time without prejudice to their care. It will be documented whether or not each subject completes the clinical study. If the Investigator becomes aware of any serious, related adverse events after the subject completes or withdraws from the study, they will be recorded in the source documents and will be reported as an adverse event to the IRB.

Clinicians and clinical staff can withdraw their participation from the interviews at any time without specific reason.

4.4.1 Early Termination Study Visit

For the intention to treat analysis, any parent who withdraws or drops out of the study will be considered as a smoker.

5 STUDY EVALUATIONS AND MEASUREMENTS

5.1 Screening and Monitoring Evaluations and Measurements

5.1.1 Medical Record Review

The following variables will be abstracted from the EHR to measure the sustainability of the intervention. The relevant domains the items address are listed parenthetically:

- Parental smoking status (Ask)
- Child’s tobacco smoke exposure at home (Ask)
- NRT prescription given/referred to BrightMedical (Advise and Refer)
- Referred to the Quitline (Refer)
- Referred to the SmokefreeTXT program (Refer)
- Other smoking or vaping related information collected in the EHR
- Parent and child health and demographic characteristics (e.g., child’s insurance status, child’s race/ethnicity, child’s age, as well as all diagnosis, treatment, updated contact information and health care services utilization)

5.1.2 Laboratory Evaluations

- Salivary cotinine swab will be taken from parents who report quitting smoking at the 12-month follow-up time period. The participant's Study ID number will be used to identify saliva samples sent to the laboratory for the cotinine analysis. No identifying information will be given to the testing laboratory.

- Parents who reported using NRT or e-cigarettes or have cotinine levels ≥10 ng/ml will have the option to get carbon monoxide levels tested to confirm combusted tobacco abstinence, to confirm self-reported smoking cessation. We will ensure that we utilize the leading infection control technologies and practices for any sample collection.
    - For in-office follow ups, to prevent missing parents who might be eligible for carbon monoxide test, we may ask parents who report current use of NRT or e-cigarettes to get their carbon monoxide levels tested in-office without knowing their cotinine levels yet. We will use their carbon monoxide test readings only if their cotinine levels are ≥10 ng/ml (we will get the cotinine results from the lab).

5.1.2.1 Laboratory Evaluations

1. **Cotinine**

To confirm cessation at 12 months, parents will complete a salivary cotinine specimen either collected in the clinic with a study team member or collected remotely. In the latter case, collection will be performed in front of study team member via a live-video conference, whenever possible.

- Subjects will use a salivary swab measurement that they are able to perform on their own that needs to be mailed to the study team/lab after sample collection.

- This will be performed either in front of a study team member at the primary care clinic or in front of a study team member via a video-conferencing session, if possible. No videos will be recorded. The specimen will be mailed by the respondent in a pre-stamped envelope to the lab.

We will use a feasible system for salivary cotinine collection that enables study participants to easily obtain their own saliva sample and does not require refrigeration. The obtained saliva samples will be sent to laboratory for cotinine testing. The RA will explain the procedures in detail before and after obtaining the cotinine swab. The sample will be

obtained in a quiet, discreet manner in the most private location possible in the practice/CHOP setting. Samples will be subsequently sent to laboratories for analysis. The laboratory will send the results via email to the study staff. If requested by the parent, the results of the cotinine test will be shared with them. Parents who will consent and provide the sample will be given a $50 incentive. The sample will remain anonymous by identifying it with the parent's study ID number.

Parents who do not have a return annual visit or a follow-up visit during the follow-up time-period (or if there are in-person visit restrictions due to COVID-19), may be contacted remotely for the 12-month survey; parents reporting 7-day abstinence will receive a saliva collection kit with instructions by mail. When possible, saliva sample collection will be performed in front of study team member via a live-video conference call. No videos will be recorded.

2. **Carbon monoxide (CO)**

In this study, in addition to the cotinine test, parents reporting 7-day abstinence and who also report current use of NRT or e-cigarettes or have cotinine levels ≥10 ng/ml will have the option to get carbon monoxide levels tested to confirm combusted tobacco abstinence. The breath CO test is non-invasive and an easily administered test. RAs will obtain breath samples from all eligible parents who agree to be tested using breath CO monitors.

For in-office follow ups, to prevent missing parents who might be eligible for carbon monoxide test, we may ask parents who report current use of NRT or e-cigarettes to get their carbon monoxide levels tested in-office without knowing their cotinine levels yet. We will use their carbon monoxide test readings only if their cotinine levels are ≥ /ml (we will get the cotinine results from the lab).

CO breath levels will be measured using the CO breath analyzer according to the manufacturer's recommendations. Parents will be asked to hold their breath for 20 seconds and then exhale slowly and fully into the mouthpiece of the instrument during which CO breath was recorded. The CO breath levels are given in parts per million (ppm). The device will be calibrated according to the manufacturer's instructions prior to use. For CO confirmed quitting, expired-air CO cutoff level of < 5ppm will be considered quit.

The single use mouthpieces are individually wrapped for ease of handling and greater infection Mouthpieces will be replaced after every use or an individual device will be used per parent. The wipes provided with the breath monitors have been tested by the supplier and found effective against (COVID-19/SARS-COV-2), as the product shows efficacy against feline coronavirus, a surrogate coronavirus (for COVID-19/SARS-COV-2).

Those parents who do not have a scheduled visit during the follow-up time-period, may be contacted remotely for the 12-month survey; parents reporting 7-day abstinence and on NRT or e-cigarettes, or who have a cotinine level of ≥10 ng/ml will be offered to get carbon monoxide levels tested to confirm cessation. They will receive a CO monitor in the mail with instructions. The results can be uploaded in real-time by the parent via a smartphone app and when possible, breath test will be performed in front of study team member via a live-video conference call. If a parent does not have a smartphone, they will be able to communicate the results back via a call or text to a study team or any other communication approach that is most convenient to them.

For remote follow ups, to minimize the burden of data collection, we will aim to send the CO monitors to eligible parents after we get their cotinine results back. If this strategy impedes the collection of CO readings, we may consider sending the CO monitors and cotinine kits simultaneously but will still use the CO readings only of the cotinine level is ≥10 ng/ml.

Parents who will consent and provide the breath sample (in the clinic or remotely) will be given a $50 incentive. They will be compensated for CO test completion regardless of whether their CO reading will be used.

5.1.2.2 Table: Clinical Laboratory Tests

Category	Tests
Smoking Cessation Testing	1. Saliva Cotinine Test<br>2. CO Breath Test

5.2 Efficacy Evaluations

Below is the complete list of all outcomes that will be collected and evaluated throughout the study.

Table 3: Main Outcomes Measurement

Outcomes	Operational Definition and Assessment Method	Sources for Construct	Timing
**Parents' combusted tobacco quit rates, and tobacco free behaviors**	**Assessed by exit or telephone survey of parents [longitudinal cohort]**		
7-day abstinence (Primary Outcome for Aim #1)	% of parental smokers who have 7-day abstinence (biochemically verified at 12-months)	BRFSS[42]; NHIS[43]	12-months post initial visit
Quit attempts	% of parental smokers who report at least one 24-hour quit attempt since pediatric visit	BRFSS[42]; NHIS[43]	12-months post initial visit
Use and Duration of pharmacotherapy	% of parental smokers who report use of gum or patch since pediatric visit and duration of use	Winickoff et al.[44]	12-months post initial visit

Institution of smoking bans	% of parental smokers who report strict smoke-free and e-cigarette free home and car rules	Drehmer at al.[18] Winickoff et al.[44,45]Emmons et al[46]Bauman et al.[47]Ossip et al.[23]Nabi-Burza et al.[48]	12-months post initial visit
**Establish the incremental cost per quit of the intervention compared to usual care control**	**Assessed by observation and survey of personnel**		
Health care system costs: time use of IT personnel, CHN, clinician, practice staff, and training personnel	Total time devoted to intervention activities using the direct observation log (Appendix 9) multiplied by national average wage rate for each type of personnel	Neumann et al.,[49] Bureau of Labor Statistics[50]	Prior to initial visit, 12- and 24-months post initial visit, direct observation log, navigator logs
Societal costs (exploratory): Parent costs (parent time, parent out-of-pocket costs for medication), Quitline costs, medication costs	Total parent time devoted to intervention/usual care activities multiplied by national average wage rate by age; published data for Quitline and medication costs[51,52]	Neumann et al.[49] Bureau of Labor Statistics[50]	Prior to initial visit, 12- and 24-months post initial visit, navigator logs
**Assess delivery and sustainability of interventions**	**Assessed by 12-month follow-up survey of parents, iPad screener, EHR data documentation and key informant interview**		Parental 12-month follow-up survey.<br><br>Pre-visit screener data, EHR data documentation before and immediately following implementation, 6 12, 18, 24, 30, and 36 months post implementation
Delivering cessation assistance	% of parental smokers who received at least one of the following<br>1. prescription of pharmacotherapy or 2. referral to Quitline or SmokefreeTXT	HEDIS measures;[53] Winickoff et al.[44,54,55]	
Screening for parental smoking	% of all parents seen in practice who are screened for smoking status using the screener data	HEDIS measure[53]	
Advising parents to quit	% of parental smokers advised to quit smoking	HEDIS measure[53]	
Counseling parents about rules	% of parental smokers counseled about strict smoke-free and<br>e-cigarette free home and car rules	Drehmer at al.[18] Winickoff et al.[56]	
Implementation and sustainability factors	Clinician and key staff member assessment of practice change resulting from the intervention	CFIR interview guide[57] ACIC,[58–60] PSAT,[59,60] IMPROVE[61,62], RE-AIM[63]	6-weeks following implementation and at 12 and 24 months

5.2.1 Diagnostic Tests, Scales, Measures, etc.

The list of tests and measures is provided above.

5.3 Safety Evaluation

This study presents no more than minimal risk to study participants, as the interventions aim to promote adult smoking cessation, which is universally recommended evidence-based practice. Though adverse events are not anticipated and will not be formally monitored (given the minimal risk nature of the study), should any events be reported to the study team, the information will be immediately shared with the CHOP IRB. We do not anticipate any physical risks to participants. Participants may experience minor phycological discomfort when answering certain questions (e.g., about their smoking habits). In such cases they may choose to not answer these questions.

6 STATISTICAL CONSIDERATIONS

6.1 Primary Endpoint

Parental combusted tobacco quit rate at 1 year, as assessed by validated surveys, biochemically-confirmed at 12-months compared between iEHR + Navigator vs. usual care control.

- Measured by the percentage of parental smokers who have 7-day abstinence (biochemically verified at 12-months).

6.2 Secondary Endpoints

1. To compare parents' self-reported adoption of tobacco free behaviors (quit attempts, use of pharmacotherapy, use of Quitlines and institution of smoking and vaping bans in their homes and cars) between the iEHR + Navigator and usual care arms
2. To establish the incremental cost per quit of the iEHR + Navigator vs. usual care arms
3. To assess the implementation and sustainability of the intervention in the iEHR + Navigator arm (NRT prescription and Quitline referrals as assessed by the parental report around 12-months, the screening survey conducted at check-in, and EHR documentation of screening and services delivery fidelity, before and immediately following implementation, 6, 12, 18, 24, 30, and 36 months).

6.3 Statistical Methods

6.3.1 Baseline Data

The iEHR pre-visit screening questionnaires (Appendix 5 for intervention practices and Appendix 5A for control practices) will collect parental baseline smoking status.
The Parent Enrollment Survey (Appendix 4) will collect additional information about vaping status, stage of readiness to change, demographic information tobacco related attitudes and behaviors, parent's name, and contact information (~ 15 minutes). All parents enrolled will receive a handout from the Research Assistant with food insecurity resources listed.

The primary effectiveness endpoint will be to compare parents' combusted tobacco quit rates between iEHR + Navigator vs. usual care control at 12-months follow-up time. Parents lost to follow-up will be considered as smokers.

The secondary efficacy endpoint will be to compare parents' adoption of tobacco free behaviors between the iEHR + Navigator vs. usual care control arms, to establish the incremental cost per quit of the intervention arm compared to usual care control and to assess the delivery and sustainability of the intervention.

We will also explore outcomes for clinically meaningful subgroups of participants, such as dual users of combustible and e-cigarette tobacco products.

6.3.2 Safety Analysis

This research does not qualify for oversight by the Data Safety and Monitoring Board as defined by NIH guidelines. It is not necessary to establish a board for this study since the interventions aim to promote adult smoking cessation, which is universally recommended evidence-based practice. Though adverse events are not anticipated and will not be formally monitored (given the minimal risk nature of the study), should any events be reported to the study team, the information will be immediately shared with the CHOP and MGH IRBs.

6.4 Sample Size and Power

Sample size calculations are based on having at least 8 practices with a minimum of 5 clinicians per practice. Clinically important and conservative estimates of validated 12-month quit rates based on prior studies are 13.8% in the iEHR + Navigator group and 7% in the usual care control group. These estimates are based on the worst-case scenario from prior studies where all enrolled parents lost to follow-up were assumed to be smokers at 12-month follow-up. The effective sample size needed for the primary outcome accounts for clustering at the clinician and practice level (4 practices per arm x 5 clinicians per practice) with intra-class correlation of .014 calculated from our previous research. By enrolling approximately 100 parents in each practice (400 per arm), we will have 80% power for the primary outcome (iEHR + Navigator vs. usual care control). Effect sizes for outcomes other

than cessation will likely be much greater; the study will have adequate power to detect these differences, shown in our prior studies.

7 STUDY INTERVENTION

7.1 Description

The iEHR part of the intervention arm consists of an innovative electronic platform for screening and automated support of the smoking cessation workflow to parents who smoke. iEHR will include automated EHR-generated NRT prescription/referral to BrightMedical, automated enrollment in the free Quitline and SmokefreeTXT program, and a brief motivational message delivered by the clinician (as prompted by the EHR).

Additional support of CHNs will be offered to all parents in the intervention arm. These CHNs will work with offices to provide customized smoking cessation support to household tobacco users through phone follow-up, ensuring access to medications and services, and/or home visits, depending on the needs of each family and as study resources allow. See a detailed description of the intervention and its components under *section 4.2.1.*

7.1.1 Treatment Compliance and Adherence

N/A

8 SAFETY MANAGEMENT

8.1 Clinical Adverse Events

Clinical adverse events (AEs) will be monitored throughout the study. This is described in 9.4.2.

8.2 Adverse Event Reporting

Since the study procedures are not greater than minimal risk, serious AEs (SAEs) are not expected. If any unanticipated problems related to the research involving risks to subjects or others happen during the course of this study (including SAEs) they will be reported to the IRB in accordance with CHOP IRB SOP 408: Unanticipated Problems Involving Risks to Subjects. AEs that do not meet prompt reporting requirements will be tracked and documented internally by the study team but not submitted to the IRB (as continuing reviews are not required). This is described in 9.4.2.

9 STUDY ADMINISTRATION

9.1 Treatment Assignment Methods

9.1.1 Randomization

In this 2-arm cluster randomization of practices to ‘iEHR +Navigator’ and ‘usual care control’ arms, we will recruit at least four matched pairs of practices through the CHOP network. Practices will be matched with respect to size and Medicaid rate. Within each of these pairs of practices, we will assign one to the intervention and the other to control, using computer generated random allocation. When the four assignments are complete, a fifth computer-generated random number will assign either "A" or "B" to the intervention. The statisticians will be blinded as to which of "A" and "B" is the intervention until the analyses are completed. In the event that a practice drops out, we will replace it with the most similar practice based on practice size and Medicaid rate from the pool of unassigned and interested practices. In the event the enrollment is slower than projected, we will add additional practices from the CHOP network, with practices matched in pairs based on practice size and Medicaid rate, from the pool of unassigned and interested practices. We will randomly allocate these practices in pairs to the control and intervention arms, as needed and in decreasing order of projected enrollment rate.

9.1.2 Blinding

The practices in each pair (the four initial pairs, and any subsequent pairs added in the event of slow accrual) will be randomly assigned labels "A" and "B". The intervention will be blinded in a separate randomization with either the label "A" or the label "B", and the other label will be assigned to the control practice. The statisticians will be blinded as to which of "A" and "B" is the intervention until the analyses are completed.

9.1.3 Unblinding

After the analyses are completed, the arms will be unblinded.

9.2 Data Collection and Management

The plan will be consistent with CHOP Policy A-3-6: Acceptable Use of Technology Resources that defines the requirements for encryption and security of computer systems. Only investigators and trained staff will have access to study information, both raw data and machine-readable files. The final dataset that will be used for the analyses will be HIPPA-limited (e.g., containing birth dates, dates of service, zip codes). Identifiable patient health information collected will also be managed in accordance with 42 CFR Parts 160 and 164 (HIPAA) federal regulations pertaining to the privacy of patient-related health information. All investigators and research staff will be HIPAA and human subjects research certified through the completion of web-based human subjects protection training, such as the CITI course. Additionally, the computer systems at the MGH and CHOP containing confidential data will have a level and scope of security that equals or exceeds those established by the Office of Management and Budget (OMB) in OMB Circular No. A-130 - Security of Federal Automated Information Systems.

9.2.1 Baseline Survey and 12-months Follow-up Data

At baseline, parents will be screened for enrollment eligibility through a review of patient medical records. Eligible parents will be offered enrollment in the study. If a parent agrees, they will be consented using Informed Consent of Parent (Appendix 3 (for intervention group) and 3A (for control group)) and they will complete the Parent Enrollment Survey (Appendix 4) by the RA. The Parent Enrollment Survey (Appendix 4) will collect parental vaping status, stage of readiness to change, demographic information, tobacco related attitudes and behaviors, their name, and contact information (~ 15 minutes). All the data will be collected via REDCap and shared between MGH and CHOP. The list of parents enrolled in the intervention arm will be shared with the CHN at CHOP.

Figure 2: Study Data Flows

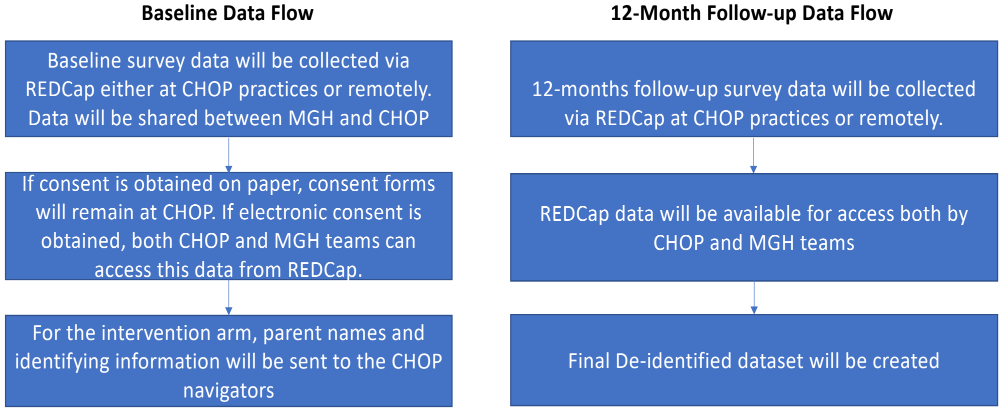

9.2.2 Data sharing with the Quitline

For parent participants consented into the longitudinal cohort (Aims 1 & 2), the generated reports that contain parent names and contact information, used to facilitate the clinical referral process to the Quitline, will be stored on a secure HIPAA-compliant server managed by CHOP. We will use appropriate secure mechanisms with authentication for transferring these reports between the research team and the Quitline vendor. The Quitline will add information about parent smokers who talk with the Quitline, are unreachable by the Quitline, and enroll or do not enroll in treatment to the reports. These reports will then be securely sent back to the research team. The reports will contain participants' identifiable information (e.g., name, phone number), so that the study team can link them back with other study-related information. These reports will be password protected and will not be stored on portable media devices (e.g., flash drives). Access to these reports will be restricted to appropriately authorized members of the study team. These reports will be transferred between CHOP and MGH teams via a secure HIPAA-compliant file transfer.

9.2.3 Data Sharing with the SmokefreeTXT

For parent participants consented into the longitudinal cohort (Aims 1 & 2), parent contact information and preference to enroll into the SmokefreeTXT program will be stored on a secure HIPAA-compliant server managed by CHOP. We will use appropriate secure mechanisms with authentication for transferring this information to the SmokefreeTXT vendor. The SmokefreeTXT program will provide information about parent smokers' interaction with the program and securely transfer it back to the research team. These reports will contain participants' identifiable information (e.g., name, phone number), so that the study team can link them back with other study-related information. These reports will be password protected and will not be stored on portable media devices (e.g., flash drives). Access to these reports will be restricted to appropriately authorized members of the study team. These reports will be transferred between CHOP and MGH teams via a secure HIPAA-compliant file transfer.

9.2.4 Data Sharing with the BrightMedical

For parent participants consented into the longitudinal cohort (Aims 1 & 2), parent contact information and preference to get the NRT medication will be stored on a secure HIPAA-compliant server managed by CHOP. We will use appropriate secure mechanisms with authentication for transferring this information to the BrightMedical, NRT delivery vendor. The BrightMedical program will provide information about parent smokers' interaction with the program and securely transfer it back to the research team. These reports will contain participants' identifiable information (e.g., name, phone number), so that the study team can link them back with other study-related information. These reports will be password protected and will not be stored on portable media devices (e.g., flash drives). Access to these reports will be restricted to appropriately authorized members of the study team. These reports will be transferred between CHOP and MGH teams via a secure HIPAA-compliant file transfer.

9.2.5 Data transfer of the EHR extractions between CHOP and MGH

9.2.5.1 Parent-level outcomes (Aims 1 & 2)

For parent participants consented into the longitudinal cohort (Aims 1 & 2), the research team will securely extract electronic health record (EHR) data from CHOP. The data will be linked with other study-related information, including surveys, engagement with Quitline, SmokefreeTXT, BrightMedical, the navigators, NRT prescriptions, child's tobacco exposure at home, parental smoking status, parent and child health and demographic characteristics (e.g., child's age, race, diagnosis), and other smoking or vaping related information. The datasets will contain participants' identifiable information (e.g., name, phone number). These datasets will be password protected and will not be stored on portable media devices

(e.g., flash drives). Access to these datasets will be restricted to appropriately authorized members of the study team. These datasets will be transferred between CHOP and MGH teams via a secure HIPAA-compliant file transfer.

9.2.5.2 Practice-level outcomes (Aim 3)

For practice level outcomes (Aim 3), we will generate a HIPAA-limited dataset (e.g., containing birth dates, dates of service, zip codes) looking at the clinic level assessment of intervention implementation and sustainability. As part of this assessment, we will extract information on engagement with Quitline, SmokefreeTXT and BrightMedical referrals, NRT prescriptions, child's tobacco exposure at home, parental smoking status, parent and child health and demographic characteristics (e.g., child's age, race, diagnosis), and other smoking or vaping related information. Provider and practice names will be coded, and separate crosswalks will be maintained only at CHOP. These HIPAA-limited reports will be transferred between CHOP and MGH teams via a secure HIPAA-compliant file transfer.

9.2.6 Data Sharing with the ADA Transcription

Clinicians and staff interviews will be transcribed. Recordings will be uploaded to the transcription service provider (ADA Transcriptions) by CHOP. ADA is a HIPAA-compliant, full-service transcription company specializing in medical and academic research. The audio-recordings will be uploaded and temporarily stored on ADA Transcriptions online platform with encryption and HIPAA compliant server. ADA Transcriptions will de-identify and complete the transcriptions and provide each audio-recording and transcription a filename and password-protect it. Only specific personnel from ADA transcriptions have access via trackable password-protected logins to the online platform system. ADA transcriptions will only temporarily store recordings and transcriptions. All data retained by ADA Transcriptions will be destroyed after final payment is received, and once transcripts are securely transferred to designated study team. This includes hard drives, back up hard drives, and any and all device copies that may exist. The password protected transcriptions will then be securely transferred to the designated research team listed on the online platform system. Only designated CHOP and MGH study team staff will be given access to transcriptions.

9.2.7 Confidentiality

Comprehensive measures will be implemented to maintain subject confidentiality as appropriate. Data will be collected using REDCap (Research Electronic Data Capture) as a data collection tool. REDCap is a web-based application developed by Vanderbilt University to capture data for clinical research and create databases and projects. It is Health Insurance Portability and Accountability Act (HIPAA)–compliant, highly secure, and intuitive to use. Data collected using the surveys via REDCap will be identifiable when it is received at MGH and CHOP teams. This is necessary so that data quality can be monitored, for CHNs

to contact the enrolled parents in the intervention arm and to complete the 12-month follow-up survey.

Every parent participant enrolled in the study will be identified by a Study ID number. The Study ID number for each subject will be a unique combination of practice and participant identifiers. The practice component of the number will be assigned to the practice when it enters the study. Personal identifiers or identifiable data will be used by the CHNs to contact the parents who enrolled in the study and by the study staff when conducting the 12-month follow-up survey.

During data collection phase, all enrollment data will be securely stored in REDCap database. Both CHOP and MGH teams will have access to the REDCap database. Data collection will be closely monitored daily by the study staff. All research assistants will be trained to explain the purpose of the study in 1-2 sentences and assure confidentiality of respondents' comments and voluntariness of their participation, all in less than a minute. The RA's will be trained to show the screen with the survey to the participant, so that the participant can point out the answers if they would like to do that. Whenever possible, the surveys will be conducted in a private area where other people cannot hear the answers of the respondents. If due to COVID parent screening and enrollment have to be modified to kiosk/telephone/remote screening, all the information will still be securely stored in the REDCap database and the procedures outlined below for the telephone surveys will apply.

Similarly, strict data confidentiality measures will be followed when conducting 12-month follow-up with the longitudinal cohort. In addition to calls to subjects by the team, parents will also be given the option of calling the researchers collect or at a toll-free number at their convenience to complete the telephone surveys. Many questions will allow yes/no responses so as to minimize the risk of accidental disclosure of potentially sensitive information. Parents will be assured that their responses will not be shared with anyone at the practice. Study subjects will have the right to discontinue the survey at any time and will be informed of this before starting the questionnaire.

Every clinician participant enrolled in the study will be identified by a Practitioner ID number. The clinicians and staff at the participating intervention practices who will participate in in-depth qualitative interviews 6-weeks following implementation and at 12, and 24 months will complete consent forms (Appendix 8). These consent forms will contain information about the study and about the interviews and the study. The completed paper consent forms will be kept in a locked location at CHOP and kept in a locked location at CHOP for 10 years and then destroyed. Electronic parent and clinician consent forms will be stored securely on the REDCap server for 10 years and then destroyed.

All electronic data transferred to MGH will be stored on MGH HIPPA-compliant shared drives that only study staff have access to using their MGH login and password.

The final dataset that will be used for the analyses will be HIPPA-limited (e.g., containing birth dates, dates of service, zip codes). After the analyses are completed and the study is over the datasets will be de-identified. Personal identifiers other than Practitioner or Patient ID numbers will be removed from the data files. The dates (e.g., visit dates) will be altered so they can no longer be identifiers.

While the analytical datasets will be stripped off identifiers (as described above), in accordance with CHOP policy, we will retain paper and electronic raw data with identifiers for at least 6 years.

Any paper forms (e.g., paper consent forms and paper logs) will be kept in a locked location at CHOP for approximately 10 years and then destroyed.

Study consent forms that contain participant identifiers, such as name, will not contain any links to the dataset after the study is completed and project data, including electronic files, is archived.

During consenting, the study participants will be informed of the steps that will be taken to maintain confidentiality.

9.2.7.1 Interviews

The interviews will be conducted in person, by phone, or HIPPA-compliant video conferencing. The qualitative researcher conducting interviews will begin each interview by asking permission to record, outlining the purpose of the interview, describing the rights of respondents to stop the interview or recording at any time, and giving a brief explanation of how interview data will be used. Interviews will only be recorded with permission.

Recordings will be uploaded to the transcription service provider (ADA Transcriptions). ADA Transcriptions is a HIPAA-compliant, full-service transcription company specializing in medical and academic research. Additionally, CHOP has a Master Business Associate agreement with the ADA Transcription. The audio-recordings will be uploaded and temporarily stored on ADA Transcriptions online platform that is encrypted and HIPAA compliant. ADA Transcriptions will de-identify and complete the transcriptions and provide each audio-recording and transcription a filename and password-protect it. The password protected transcriptions will then be securely transferred to the designated research team listed

on the online platform system. Only designated CHOP and MGH study team staff will be given access to transcriptions. The interview data will be used anonymously in conference presentations, articles, and reports. As part of the rapid-response quality improvement, data will also be used to improve the intervention and address factors that influence implementation of the intervention.

The transcripts from the interviews will be stored on a secure server, with access limited to the researchers who are working with the data.

Transcripts will be de-identified as much as possible. This will be done by providing guidance to the interviewees during the introduction to avoid using identifiers, by using pseudonyms, removing location names, and/or removing other data that could identify the practice, respondent, or location.

9.2.7.1.1 Use and storage

The de-identified transcripts will be used for coding and analysis.
When and if needed, the de-identified transcripts will be shared between study team researchers at different institutions. These transcripts will be shared via secure file transfer.
The sound files and the transcriptions will be stored electronically; the files will be password protected for approximately 10 years and then destroyed.

While the research team will make every effort to maintain practitioner confidentiality, it cannot be absolutely guaranteed. Risks will be reduced by deleting practitioner names from all study data after data collection is complete and replacing them with practitioner identifier numbers, as well as password protecting files. Records that identify them and their signed consent forms may be inspected by a regulatory agency (the Department of Health and Human Services), MGH and/or CHOP.

9.2.8 Security.

Back-up of the dataset will be saved on the MGH and CHOP's secure servers.

9.2.9 Anonymization, de-identification or destruction.

The final dataset that will be used for the analyses will be HIPPA-limited (e.g., containing birth dates, dates of service, zip codes). After the analyses are completed and the study is over the datasets will be de-identified. Personal identifiers other than Practitioner or Patient ID numbers will be removed from the data files. The dates (e.g., visit dates) will be altered so they can no longer be identifiers. While the analytical datasets will be stripped off the identifiers, in

accordance with CHOP policy, we will retain paper and electronic raw data with identifiers for at least 6 years. Identifiable patient health information collected will also be managed in accordance with 42 CFR Parts 160 and 164 (HIPAA) federal regulations pertaining to the privacy of patient-related health information. All investigators and research staff will be HIPAA and human subjects research certified through the completion of web-based human subjects protection training, such as the CITI course. Additionally, the computer systems at the MGH and CHOP containing confidential data will have a level and scope of security that equals or exceeds those established by the Office of Management and Budget (OMB) in OMB Circular No. A-130 - Security of Federal Automated Information Systems.

The results of this research study may be presented at meetings or in publications but will be reported in summary form only.

Study consent forms that contain participant identifiers, such as name, will not contain any links to the dataset after the study is completed and project data, including electronic files, is archived.

All study data that resides on the CHOP server will be saved electronically for 10 years and then destroyed. All data collected on paper (e.g., consent forms, paper logs) will be kept in a locked cabinet at CHOP and will be destroyed 10 years following the study end date.

9.3 Confidentiality

No identifiable data will be used for any future studies.

9.4 Regulatory and Ethical Considerations

9.4.1 Data and Safety Monitoring Plan

This is a minimal risk clinical trial (as defined in federal regulation at 45 CFR 46.102(i)). This trial aims to study the effectiveness of a tobacco control interventions iEHR + Navigator in the pediatric office setting to help household members of children seen at the office quit smoking. Enrollees will be given information on how to contact the Principal Investigator (Dr. Fiks) and the MGH Lead Investigator (Dr. Winickoff) to report any study related problems. Drs. Fiks and Winickoff will also work closely with research staff to ensure that all data are being stored in password-protected systems and that data collection, storage and transfer follow the safety and confidentiality procedures outlined in this protocol. CHOP PI (Dr. Fiks) will monitor and review the study progress and the accuracy and security of the emerging data at CHOP. Under the NIH policy on the use of a single IRB for multi-site research, the Children's Hospital of Philadelphia (CHOP) Institutional Review Board (IRB) will serve as the reviewing IRB for this study. All participating sites will

adhere to the sIRB Policy (https://grants.nih.gov/grants/guide/notice-files/NOT-OD-16-094.html). Massachusetts General Hospital's (MGH) IRB has agreed that CHOP will serve as the sIRB. The CHOP PI and MGH Lead Investigator will work closely with the CHOP and MGH IRBs for the duration of the study to ensure the safety of the interventions.

9.4.2 Risk Assessment

Though adverse events are not anticipated (given the minimal risk nature of the study), should any events occur, they will be reported to the study team immediately and shared with all relevant IRBs. The CHOP PI, MGH Lead Investigator, study coordinators, and all members of the research staff are responsible for the assessment and reporting of adverse events to the IRB. Weekly reports of practice and parent recruitment will be monitored by MGH and CHOP staff. All spontaneous reports by subjects, observations by clinical research staff, and reports to research staff by family or healthcare clinicians will be investigated by the Steering Committee, as further described in the section C1 of the research narrative. In case of an adverse event, all information related to the event will be immediately shared with all relevant IRBs. The investigators will assess the relationship of the adverse event as not related, possibly related, probably related, or definitely related to the intervention; this assessment will be done with standard criteria for clinical trials.

A possible adverse event (to qualify, the adverse event must meet 2 of the following conditions):

1. has a reasonable temporal relationship to the intervention
2. could not readily have been produced by the subject's clinical state
3. could not readily have been due to environmental or other interventions
4. follows a known pattern of response to intervention
5. disappears or decreases with reduction in cessation of intervention.

Probably related to the intervention (to qualify, the adverse event must meet 3 of the following conditions):

1. has a reasonable temporal relationship to the intervention
2. could not readily have been produced by the subject's clinical state
3. could not readily have been due to environmental or other interventions
4. follows a known pattern of response to intervention
5. disappears or decreases with reduction in cessation of intervention.

Definitely related to the intervention (to qualify, the adverse event must meet at least 4 of the following conditions):

1. has a reasonable temporal relationship to the intervention
2. could not readily have been produced by the subject's clinical state
3. could not readily have been due to environmental or other interventions

4. follows a known pattern of response to intervention
5. disappears or decreases with reduction in cessation of intervention.

9.4.2.1 Adverse Event Definitions

Adverse Event: an undesirable and unintended result of therapy, intervention or interaction experienced by a subject participating in a research study.

Unexpected Adverse Event: any adverse event, the specificity, severity, frequency or nature of which is not consistent with the current general investigational protocol or investigational protocol amendments.

Serious Adverse Event: any adverse event that results in any of the following outcomes: death, a life-threatening adverse event, inpatient hospitalization or prolongation of existing hospitalization, a persistent or significant disability/incapacity, or a congenital anomaly/birth defect. Important medical events that may not result in death, may not be life threatening, or may not require hospitalization could be considered serious adverse events when, based upon appropriate medical judgment, they may jeopardize the patient or subject and may require medical or surgical intervention to prevent one of the outcomes listed in this definition. Due to the minimal risk nature of this intervention, in which we collect data only through surveys, we do not expect serious adverse events.

9.4.2.2 Adverse Event Reporting

An FDA Medwatch Form will be used to report all Adverse Events. A copy of the adverse event report will be retained with the subject's research records.

Serious Adverse Events must be reported in writing within 7 calendar days of any member of the investigative team becoming aware of such an event. These adverse events would be reported to NCI and to the CHOP and MGH IRBs.

Unexpected Adverse Events must be reported in writing within 15 calendar days of any member of the investigative team becoming aware of such an event.

9.4.2.3 Management of Reported Adverse Events:

The CHOP PI, MGH Lead Investigator and the study team are responsible for the appropriate clinical management of all adverse events. The CHOP PI and MGH Lead Investigator will ensure that all appropriate resources are directed toward subject safety and well-being. Any subject may discontinue the study at any time at their own discretion or if in the opinion of any study staff, their safety or well-being is jeopardized by continued participation in the study. If any unanticipated problems related to the research involving risks to subjects or others happen during the course of this study (including SAEs) these will

be reported to the IRB in accordance with CHOP IRB SOP 408: Unanticipated Problems Involving Risks to Subjects. AEs that do not meet prompt reporting requirements will be tracked and documented internally by the study team but not submitted to the IRB (as continuing reviews are not required). Otherwise, subject enrollment, project status, adherence data will be discussed among the scientific team. A statement reflecting the results of ongoing data review will be submitted annually to NIH or as requested in accordance with contractual requirements.

9.4.3 Potential Benefits of Trial Participation

Carrying out this research in the pediatric setting will likely increase the provision of tobacco control services for parents who smoke. Increased provision of services has been proven to increase the chances of changing smoking behaviors. Parents who successfully quit or reduce their tobacco consumption may live longer with a better quality of life, reduce the tobacco smoke exposure of their spouses, children, and others, and have increased financial resources. Mothers who quit successfully may be non-smokers for subsequent pregnancies, leading to better outcomes for their unborn children. The children of parents who quit smoking are less likely to grow up to be smokers themselves. However, these benefits cannot be guaranteed to all parents and families who participate in the research.

In addition to the potential benefits to participants, the benefits of this study will include improvement of science and clinical practice. The results will also help to develop and implement fully integrated, cost-effective, disseminable, and sustainable strategies to optimize parental smoking cessation outcomes in pediatric settings on a national scale. The minimal risks of participation are reasonable in relation to the potential benefits of these interventions. It is also possible that the information obtained from this project will be used to develop clinician guidelines and education, which could directly benefit the study subjects in the future.

9.4.4 Risk-Benefit Assessment

Risks to participants will be minimal and include discomfort in discussing personal tobacco use with a stranger, and the receipt of tobacco-related services from their child's clinician. Study participants will lose approximately ~ 15 minutes of time that they could have used for other activities. In addition, subjects will be asked to participate in the longitudinal cohort survey 12 months after the index visit, which will take approximately 15 minutes at the practice exit or remotely and if eligible, giving a salivary sample and doing a CO breath test. The same risks apply.

Carrying out this research in the pediatric setting will likely increase the provision of tobacco control services for parents who smoke. Increased provision of services has been proven to increase the chances of changing smoking behaviors. Parents who successfully quit or reduce their tobacco consumption may live longer with a better quality of life, reduce

the tobacco smoke exposure of their spouses, children, and others, and have increased financial resources. Mothers who quit successfully may be non-smokers for subsequent pregnancies, leading to better outcomes for their unborn children. The children of parents who quit smoking are less likely to grow up to be smokers themselves. However, these benefits cannot be guaranteed to all parents and families who participate in the research.

9.5 **Recruitment Strategy**

As part of the clinical workflow, all parents in control and intervention practices will be asked about their individual smoking status on the iEHR pre-visit screening questionnaire (Appendix 5 for intervention practices and Appendix 5A for control practices).

The study team/CHOP Clinical Reporting Unit (CRU) and/or Research Enhancement Core (REC) will generate rosters of potentially eligible parents for the upcoming visit. Rosters will include all upcoming visits at study practices of parents who answered, “yes” to either of the screening questions: *“Have you smoked a cigarette, even a puff, in the past 7 days?” and “Have you smoked any other tobacco product (cigars like black and mild, hookah), even a puff, in the past 7 days?”* on the iEHR pre-visit screening questionnaire. The study team will approach these potentially eligible parents to offer enrollment in the study.

Additionally, at participating CHOP primary care sites, study team will also review the medical records to identify parents who self-reported as smokers (answered “yes” to either of the screening questions: *“Have you smoked a cigarette, even a puff, in the past 7 days?” and “Have you smoked any other tobacco product (cigars like black and mild, hookah), even a puff, in the past 7 days?”* on the iEHR pre-visit screening questionnaire. (Appendix 5 for intervention practices and Appendix 5A for control practices) to approach them and offer enrollment in the study longitudinal cohort.

Potentially eligible families who are missed in the CHOP primary care sites or are not able to complete enrollment survey in the office will also be reached via recruitment emails (Appendix 12 (for intervention) and 13(control)), text messages and calls facilitated by the study team and/or CHOP Research Enhancement Core (REC).

We will deploy several strategies to maximize recruitment. Parents will be recruited in one of these ways: (1) In-office enrollment; (2) Hybrid enrollment: from in-office to remote; (3) Remote enrollment. The detailed description of each approach and overall recruitment strategy is described in detail under section 4.1. Screening & Enrollment.

Using these strategies, we will continue to enroll parents until approximately 100 parents who use combusted tobacco have been enrolled in the longitudinal cohort in that practice or approximately 400 parents who use combusted tobacco are enrolled in each arm. If some

practices accrue subjects at a rate which makes it unlikely that these practices can achieve the recruitment goal of approximately 100 subjects, then we will estimate the likely shortfall, and increase the recruitment goals in faster-accruing practices in order to attempt to preserve statistical power. If enrollment is slower than projected in some practices, we will add new practices that are similar to each other based on practice size and Medicaid rate in each arm to reach the goal of 400 parents in each arm.

A notice may be posted at the practice entrance/registration window/waiting room of participating practices, informing parents accompanying their children to office visits that the practice is participating in the research study (posting details may be modified based on practice layout and preferences). Recruitment procedures will be identical across both arms.

9.6 Informed Consent/Assent and HIPAA Authorization

For in-person parent consent, the trained research assistants at the practices will approach eligible participants (screened through the review of medical records) and ask whether they want to participate in the study. They will be given the appropriate consent documents at this time to read prior to completing any parent enrollment surveys. REDCap will be the primary method of obtaining consent from participants. Research Assistants will provide tablets with the REDCap eConsent, and participants will be provided with a copy of the signed document via email. The eConsent forms were reviewed and authorized for use by the CHOP REDCap team. In case of a tablet failure/unavailability, paper consent forms will be used. For paper consent, trained research assistants will document consent by obtaining the participant’s signature on the previously approved IRB consent document.

For remote parent consent, consent will be obtained electronically utilizing the REDCap interface, accessed by a link emailed or texted to the participant. When consent is obtained electronically, participants will be able to read, digitally sign their name and receive an emailed copy of the informed consent/HIPAA authorization form. The research presents minimal risk to participants and involves no procedures for which written consent is normally required outside of the research context. When parent outreach is performed via a phone call, a study team member will be able to answer any questions about consent in real time. Study team’s phone number and e-mail address will be included in all e-mail and text communication with parents.

The parent consent form (Appendix 3 and 3b (paper) and 3E and 3bE (electronic) will describe the risks, benefits, and rights of participation, as well as an assurance of confidentiality, including the procurement of a federal Certificate of Confidentiality. The study staff will review the documents with the family and answer any questions and give them time to decide if they want to enroll in the study. For electronic consent, the parent will be asked to electronically sign the REDCap consent form if they elect to participate and will be provided with a copy of the signed consent via email. In case of paper consent, the parent will be asked to sign and return

the appropriate consent forms if they elect to participate, retaining a copy of each form for their own records.

In intervention practices, in-depth qualitative interviews will be conducted with practice clinicians and staff 6-weeks following implementation and at 12, and 24 months.
The clinician and staff consent form (Appendix 8) will describe the purpose of the interviews, risks, benefits and rights of participation, as well as an assurance of confidentiality, including the procurement of a federal Certificate of Confidentiality. The completed paper consent forms will be kept in a locked location at CHOP and kept in a locked location at CHOP for 10 years and then destroyed. Electronic clinician consent forms will be stored securely on the REDCap server.

The consent form will be a combined consent-authorization document.

9.6.1 Waiver of Consent

n/a

9.6.2 Waiver of HIPAA Authorization

For parent subject, we incorporated a HIPPA Authorization form in the Consent Form using appropriate consent templates (Parent Consent Forms Appendix 3, 3b, 3bE, 3E).

For clinician and staff participants of the qualitative interviews we would like to request a waiver of HIPPA Authorization as we will not be collecting any protected health information for these subjects.

We would like to request a waiver of HIPAA authorization for recruitment of parents, as the study staff will need review patient medical records to identify eligible subjects (as part of recruitment). This way we can target our recruitment only to those parents who identify as smokers.

9.7 **Payment to Subjects/Families**

9.7.1 Payments to parent for time and inconvenience (i.e., compensation)

- $20 gift card will be provided to parents upon participating in the enrollment survey at baseline
- $40 gift card will be provided to parents upon participating in the follow-up survey respectively.
- $50 gift card will be provided to parents who provide a salivary sample to confirm quit status.

- $50 gift card will be provided to parents who do the CO breath test to confirm quit status.

Participants can get the gift card via e-mail, text, or in-person.

9.7.1.1 Token of Appreciation

During the navigator sessions (protocol section 4.2.2), the navigator can offer families a small item, such as a stress ball, a motivational journal, a key chain, or a coloring book, something that parents can use to distract themselves from smoking or remind them why they want to quit in the first place. CHOP community health navigators already incorporate this strategy when working with families.

9.7.2 Payments to practices for time, effort and inconvenience (i.e., compensation)

N/A

9.7.3 Payments to clinicians and staff for time, effort and inconvenience (i.e., compensation)

A $50 gift card will be given to the clinicians and staff to recognize their time and effort for participating in the qualitative interviews ($50 per interview).

10 PUBLICATION

There will be sub-committee, "**Publications, presentations, and dissemination**" which will include both MGH and CHOP members. No individually identifiable PHI will be published. Both MGH and CHOP team members will have access to the complete trial data and will work together to write papers. Both team members will discuss the papers related items and analysis plans in this sub-committee meeting.

11 REFERENCES

1. Winickoff JP, Nabi-Burza E, Chang Y, et al. Implementation of a parental tobacco control intervention in pediatric practice. *Pediatrics*. 2013;132(1):109-117. doi:10.1542/peds.2012-3901.
2. Nabi-Burza E, Drehmer JE, Hipple Walters B, et al. Treating Parents for Tobacco Use in the Pediatric Setting: The Clinical Effort Against Secondhand Smoke Exposure Cluster Randomized Clinical TrialTreating Parents for Tobacco Use in the Pediatric SettingTreating Parents for Tobacco Use in the Pediatric Se. *JAMA Pediatr*. August 2019. doi:10.1001/jamapediatrics.2019.2639.
3. Perkins KA, Karelitz JL, Jao NC. Optimal carbon monoxide criteria to confirm 24-hr smoking abstinence. *Nicotine Tob Res*. 2013. doi:10.1093/ntr/nts205.
4. Taylor SM, Ross NA, Cummings KM, et al. Community intervention trial for smoking cessation (COMMIT): changes in community attitudes toward cigarette

smoking. *Heal Educ Res*. 1998;13(1):109-122.
5. Winickoff JP, Healey EA, Regan S, et al. Using the postpartum hospital stay to address mothers' and fathers' smoking: the NEWS study. *Pediatrics*. 2010;125(3):518-525. doi:10.1542/peds.2009-0356.
6. den Exter Blokland EA, Engels RC, Hale WW 3rd, Meeus W, Willemsen MC. Lifetime parental smoking history and cessation and early adolescent smoking behavior. *Prev Med (Baltim)*. 2004;38(October 2015):359-368. doi:10.1016/j.ypmed.2003.11.008.
7. Farkas AJ, Distefan JM, Choi WS, Gilpin EA, Pierce JP. Does parental smoking cessation discourage adolescent smoking? *Prev Med (Baltim)*. 1999;28(3):213-218. doi:10.1006/pmed.1998.0451.
8. Bricker JB, Leroux BG, Peterson Jr. A V, et al. Nine-year prospective relationship between parental smoking cessation and children's daily smoking. *Addiction*. 2003;98(5):585-593.
9. Bricker JB, Leroux BG, Robyn Andersen M, Rajan KB, Peterson AVJ. Parental smoking cessation and children's smoking: mediation by antismoking actions. *Nicotine Tob Res*. 2005;7(4):501-509. doi:10.1080/14622200500186353.
10. Bricker JB, Peterson AVJ, Sarason IG, Andersen MR, Rajan KB. Changes in the influence of parents' and close friends' smoking on adolescent smoking transitions. *Addict Behav*. 2007;32(4):740-757. doi:10.1016/j.addbeh.2006.06.020.
11. U.S. Department of Health and Human Services. *The Health Consequences of Involuntary Tobacco Smoke: A Report of the Surgeon General*. Altanta, GA: U.S. Department of Health and Human Services, Centers for Disease Control and Prevention, National Center for Chronic Disease Prevention and Health Promotion, Office on Smoking and Health; 2006.
12. Winickoff JP, Berkowitz AB, Brooks K, et al. State-of-the-art interventions for office-based parental tobacco control. *Pediatrics*. 2005;115(3):750-760. doi:10.1542/peds.2004-1055.
13. Yolton K, Dietrich K, Auinger P, Lanphear BP, Hornung R. Exposure to environmental tobacco smoke and cognitive abilities among U.S. children and adolescents. *Environ Health Perspect*. 2005;113(1):98-103. http://www.ncbi.nlm.nih.gov/entrez/query.fcgi?cmd=Retrieve&db=PubMed&dopt=Citation&list_uids=15626655.
14. U.S. Department of Health and Human Services. *The Health Consequences of Smoking—50 Years of Progress: A Report of the Surgeon General*. Atlanta, GA: U.S. Department of Health and Human Services, Centers for Disease Control and Prevention, National Center for Chronic Disease Prevention and Health Promotion, Office on Smoking and Health; 2014.
15. Centers for Disease Control and Prevention. Nonfatal residential fire-related injuries treated in emergency departments--United States, 2001. *MMWR Morb Mortal Wkly Rep*. 2003;52(38):906-908.
16. Newacheck PW, Stoddard JJ, Hughes DC, Pearl M. Health insurance and access to primary care for children. *N Engl J Med*. 1998;338(8):513--9.
17. QuickStats: Cigarette Smoking Status* Among Current Adult E-cigarette Users, by Age Group - National Health Interview Survey, United States, 2015. *MMWR Morb Mortal Wkly Rep*. 2016;65(42):1177. doi:10.15585/mmwr.mm6542a7.

18. Drehmer JE, Nabi-Burza E, Walters BH, Ossip DJ, Levy DE, Rigotti NA, Klein JD WJ. Parental Smoking and E-Cigarette Use in Homes and Cars. *Accept Pediatr*. 2018.
19. Siu AL. Behavioral and Pharmacotherapy Interventions for Tobacco Smoking Cessation in Adults, Including Pregnant Women: U.S. Preventive Services Task Force Recommendation Statement. *Ann Intern Med*. 2015;163(8):622-634. doi:10.7326/M15-2023.
20. Levy DT, Graham AL, Mabry PL, Abrams DB, Orleans CT. Modeling the impact of smoking-cessation treatment policies on quit rates. *Am J Prev Med*. 2010;38(3 Suppl):S364-72. doi:10.1016/j.amepre.2009.11.016.
21. Fiore MC, Jaen CR, Baker TB, al. E. *Treating Tobacco Use and Dependence: 2008 Update. Clinical Practice Guideline*. (Service D of health, human services. Public Health, eds.). Rockville, MD: U.S. Department of Health and Human Services. Public Health Service. May 2008.
22. Rosen LJ, Noach M Ben, Winickoff JP, Hovell MF, Rosen ALJ, Noach B. Parental smoking cessation to protect young children: A systematic review and meta-analysis. *Pediatrics*. 2012;129(1):141-152. doi:10.1542/2011-0249.
23. Ossip DJ, Chang Y, Nabi-Burza E, et al. Strict smoke-free home policies among smoking parents in pediatric settings. *Acad Pediatr*. 13(6):517-523. doi:10.1016/j.acap.2013.06.003.
24. Winickoff JP, Park ER, Hipple BJ, et al. Clinical effort against secondhand smoke exposure: development of framework and intervention. *Pediatrics*. 2008;122(2):e363--75. doi:10.1542/peds.2008-0478.
25. Winickoff JP, Hipple B, Drehmer J, et al. The Clinical Effort Against Secondhand Smoke Exposure (CEASE) intervention: A decade of lessons learned. *J Clin Outcomes Manag*. 2012;19(9):414-419.
26. Winickoff JP, Berkowitz AB, Brooks K, et al. State-of-the-art interventions for office-based parental tobacco control. *Pediatrics*. 2005;115(3):750-760. doi:10.1542/peds.2004-1055.
27. Winickoff JP, Nabi-Burza E, Chang Y, et al. Sustainability of a Parental Tobacco Control Intervention in Pediatric Practice. *Pediatrics*. 2014;134(5):933-941. doi:10.1542/peds.2014-0639.
28. Winickoff JP, McMillen RC, Tanski SE, Klein JD, Weitzman M. Helping parents quit smoking with effective medications: Do child healthcare providers do what parents want? *Pediatr Res*. 2004;55(4):604A.
29. Hipple B, Nabi-Burza E, Hall N, Regan S, Winickoff JP. Distance-based training in two community health centers to address tobacco smoke exposure of children. *BMC Pediatr*. 2013;13:56. doi:10.1186/1471-2431-13-56.
30. Berwick D, Bonomi A, Curry S, Homer C, Solberg L. Personal communication interviews completed for NCI K07CA100213A01 (Winickoff PI).
31. Nabi-Burza E, Chang Y, Drehmer J, et al. Providing parental smoking cessation assistance using electronic screening. In: *Pediatric Academic Societies Annual Meeting*. Baltimore, MD; 2016.
32. Nabi-Burza E, Drehmer J, Hipple Walters B, et al. Treating Parents for Tobacco Use in the Pediatric Setting: The CEASE Randomized Control Trial. Manuscript submitted for publication. 2018.
33. Jenssen BP, Bryant-Stephens T, Leone FT, Grundmeier RW, Fiks AG. Clinical

decision support tool for parental tobacco treatment in primary care. *Pediatrics*. April 2016. doi:10.1542/peds.2015-4185.

34. Brooke J. SUS: a "quick and dirty" usability scale. In: Jordan PW, Thomas B, Weerdmeester BA, McClelland AL, eds. *Usability Evaluation in Industry*. London: Taylor and Francis; 1986.
35. Jenssen B, Muthu N, Kelly M, et al. Parent eReferral to Tobacco Quitline from Pediatric Primary Care: A Pragmatic Randomized Trial. Manuscript submitted for publication. 2018.
36. Lepore SJ, Winickoff JP, Moughan B, et al. Kids Safe and Smokefree (KiSS): a randomized controlled trial of a multilevel intervention to reduce secondhand tobacco smoke exposure in children. *BMC Public Health*. 2013;13:792. doi:10.1186/1471-2458-13-792.
37. Collins B, Lepore S, Nair U, Winickoff J, Bryant-Stephens T, Godfrey M. Improving efforts to reduce vulnerable children's exposure to tobacco smoke: Integrating health systems and individual interventions in a multilevel randomized trial. In: *American Public Health Association Annual Meeting*. Denver, CO; 2016.
38. Fiore MC, Jaen CR, Baker TB. Treating Tobacco Use and Dependence: 2008 Update. Quick Reference Guide for Clinicians. 2009. http://www.ahrq.gov/professionals/clinicians-providers/guidelines-recommendations/tobacco/clinicians/references/quickref/index.html.
39. Drehmer JE, Ossip DJ, Nabi-Burza E, et al. Thirdhand smoke beliefs of parents. *Pediatrics*. 2014;133(4):e850--6. doi:10.1542/peds.2013-3392.
40. Farber H, Walley S, Groner J, Nelson K. Clinical Practice Policy to Protect Children From Tobacco, Nicotine, and Tobacco Smoke. *Pediatrics*. 2015;136(5):1008-1017. doi:10.1542/peds.2015-3108.
41. Drehmer JE, Hipple B, Nabi-Burza E, et al. Proactive enrollment of parents to tobacco quitlines in pediatric practices is associated with greater quitline use: a cross-sectional study. *BMC Public Health*. 2016;16. doi:10.1186/s12889-016-3147-1.
42. US Department of Health and Human Services. *2000 BRFSS Summary Prevalence Report*. Atlanta, Georgia: U.S. Department of Health and Human Services, Centers for Disease Control and Prevention, National Center for Chronic Disease Prevention and Health Promotion, Division of Adult and Community Health, Behavioral Surveillance Branch; 2001.
43. Centers for Disease Control and Prevention, NCHS. National Health Interview Survey. *Cited Heal People 2010*. 1998.
44. Winickoff JP, Buckley VJ, Palfrey JS, Perrin JM, Rigotti NA. Intervention with parental smokers in an outpatient pediatric clinic using counseling and nicotine replacement. *Pediatrics*. 2003;112(5):1127-1133. http://www.ncbi.nlm.nih.gov/pubmed/14595057.
45. Winickoff JP, Friebely J, Tanski SE, et al. Beliefs about the health effects of "thirdhand" smoke and home smoking bans. *Pediatrics*. 2009;123(1):e74--9. doi:10.1542/peds.2008-2184.
46. Emmons KM, Hammond SK, Fava JL, Velicer WF, Evans JL, Monroe AD. A randomized trial to reduce passive smoke exposure in low-income households with young children. *Pediatrics*. 2001;108(1):18--24.
47. Bauman A, Strecher VJ, Greenberg RA, Haley NJ. A comparison of biochemical and

interview measures of the exposure of infants to environmental tobacco smoke. *Eval Health Prof*. 1989;12(2):179-191.

48. Nabi-Burza E, Regan S, Drehmer J, et al. Parents smoking in their cars with children present. *Pediatrics*. 2012;130(6):e1471-8. doi:10.1542/peds.2012-0334.
49. Neumann, P., Ganiats, T., Russell, L., Sanders, G., Siegel J, ed. *Cost-Effectiveness in Health and Medicine*. 2nd ed. New York: Oxford University Press; 2016. doi:10.1093/acprof:oso/9780190492939.001.0001.
50. United States Department of Labor. Bureau of Labor Statistics.
51. Hollis JF, McAfee TA, Fellows JL, Zbikowski SM, Stark M, Riedlinger K. The effectiveness and cost effectiveness of telephone counselling and the nicotine patch in a state tobacco quitline. *Tob Control*. 2007;16(Suppl 1):i53 LP-i59. doi:10.1136/tc.2006.019794.
52. Levy DE, Klinger E, Linder JA, et al. Cost-effectiveness of a health system-based smoking cessation program. *Nicotine Tob Res*. September 2016. doi:10.1093/ntr/ntw243.
53. HEDIS. *Technical Specifications. National Committee for Quality Assurance.* Washington D.C.; 2001.
54. Winickoff JP, Tanski SE, McMillen RC, Klein JD, Rigotti N a, Weitzman M. Child health care clinicians' use of medications to help parents quit smoking: a national parent survey. *Pediatrics*. 2005;115(4):1013-1017. doi:10.1542/peds.2004-1372.
55. Winickoff JP, Tanski SE, McMillen RC, Hipple BJ, Friebely J, Healey EA. A national survey of the acceptability of quitlines to help parents quit smoking. *Pediatrics*. 2006;117(4):e695--700. doi:10.1542/peds.2005-1946.
56. Winickoff JP, McMillen RC, Carroll BC, et al. Addressing parental smoking in pediatrics and family practice: a national survey of parents. *Pediatrics*. 2003;112(5):1146-1151.
57. Damschroder LJ, Aron DC, Keith RE, Kirsh SR, Alexander JA, Lowery JC. Fostering implementation of health services research findings into practice: a consolidated framework for advancing implementation science. *Implement Sci*. 2009;4:50. doi:10.1186/1748-5908-4-50.
58. Bonomi AE, Wagner EH, Glasgow RE, VonKorff M. Assessment of chronic illness care (ACIC): a practical tool to measure quality improvement. *Health Serv Res*. 2002;37(3):791-820.
59. Schell SF, Luke DA, Schooley MW, et al. Public health program capacity for sustainability: a new framework. *Implement Sci*. 2013;8:15. doi:10.1186/1748-5908-8-15.
60. Program Sustainability Assessment Tool.
61. Solberg LI, Kottke TE, Brekke ML, Conn SA, Magnan S, Amundson G. The case of the missing clinical preventive services systems. *Eff Clin Pract*. 1998;1(1):33--8.
62. Solberg LI, Kottke TE, Brekke ML. Will primary care clinics organize themselves to improve the delivery of preventive services? A randomized controlled trial. *Prev Med*. 1998;27(4):623--31.
63. RE-AIM.

APPENDIX

Appendix 1: Recruitment e-mail letter to CHOP practices
Appendix 2: Qualitative interview information Sheet
Appendix 3, 3b, 3E and 3bE: Intervention and Control practices parent consent
Appendix 4: Parent enrollment survey
Appendix 5: CEASE pre-visit screening questionnaire (Intervention)
Appendix 5A: CEASE pre-visit screening questionnaire (Control)
Appendix 6: 12-month follow-up survey
Appendix 7: Qualitative interview guides
Appendix 8: Clinician consent form
Appendix 9: Cost-effectiveness direct observation log
Appendix 10: Eligibility Form
Appendix 11: Navigator Form
Appendix 12: eCEASE INTERVENTION Recruitment Email Template
Appendix 13: eCEASE CONTROL Recruitment Email Template

Title: **Electronic Pediatric Office Systems to Support Treatment for Parental Tobacco Use**

Short Title: eCEASE

Drug or Device Name(s): N/A

NCT Number: NCT04974736

Sponsor: NIH

eIRB Number: IRB 20-018146

Version Date: 11/10/ 2020

Statistical Analysis plan.

Primary outcome: Parental 7-day quit rates, as assessed by validated surveys and biochemically- confirmed at 12-months, compared between iEHR+Navigator and usual care control arms. The study is powered for this outcome.

The primary outcome will compare the parental combusted tobacco cessation, biochemically confirmed between the intervention arm (iEHR + Navigator) vs. usual care control. For cotinine confirmed quitting, only self-reported non-smokers who have cotinine values <10 ng/ml, a consensus cutoff level, will be considered quit. Parents on NRT, or e-cigarettes, or who fail the cotinine test will be asked to have their carbon monoxide (CO) levels tested, to confirm self-reported smoking cessation. For CO confirmed quitting, expired-air CO cutoff level of < 5ppm will be considered quit. E-cigarette use will be noted and analyzed separately as the literature emerges.

In our intention-to-treat analysis, all participants lost to follow-up will be considered smokers. We will also use multiple imputation techniques, as appropriate to impute missing data. We will used generalized linear mixed-effects models (Gaussian, logistic, or Poisson, as appropriate) to compare the primary outcome between arms. To the extent allowed by the sample, we will build a logistic regression model that includes potential confounding factors, particularly those that are unbalanced between iEHR+Navigator vs usual care control groups to compare the parental 7-day quit rates. We will attempt to identify predictors of parental quitting to determine whether any of these factors modify intervention effect. To determine whether the intervention effect differs among subgroups, we will test the interaction between intervention group and these predictors. Specifically, to the extent allowed by our sample size, exploratory analyses will compare quit rates in subgroups of smokers characterized by cigarettes per day, e-cigarette use, number of smokers in the home, demographic variables (parent sex, age, race, education, child age) and treatment use. We will conduct a difference in difference analyses from baseline to follow up to evaluate the effect of the intervention on changes in smoking related factors like mean cigarettes per day and daily smoking rates.

Secondary outcomes

1. **Rates of smokefree homes and cars, use of NRT and duration of use, and quitline enrollment, as assessed by validated surveys of parents, will be compared between iEHR+Navigator and usual care control arms.** Change in parental smoking inside homes and cars, use of NRT, and receiving services from cessation programs will be based on the baseline and 12-months enrollment visit, comparing change from baseline to 12 months between intervention groups and usual care control practices. We will follow a similar analysis strategy to that stated above in primary outcome analysis.

2. **Establish the incremental cost per quit of intervention.** The healthcare system's perspective is central to establishing the interventions' disseminability and sustainability. As a secondary analysis, we will consider costs from a societal perspective to pave the way for future cost-utility analyses. We will calculate the incremental cost per quit of the iEHR+Navigator intervention relative to usual care, as we have done in prior studies: (Total cost at 12 months $_{arm[i]}$-Total costs $_{arm[j]}$)/(Total biochemically confirmed quits at 12 months $_{arm[i]}$-Total biochemically confirmed quits at 12 months $_{arm[j]}$). Development costs for iEHR will not be included in primary analyses as the iEHR will be fully developed and ready for dissemination by the time the intervention begins. Direct costs include iEHR set-up, customizing, implementing, and sustaining the systems changes that support service delivery and cessation, electronic tablets, and related clinician work. Direct costs further include: the CEASE intervention staff time to support the practice leader and the time clinicians and office staff take to learn and use the online module. Time use will be tracked by administrative records, direct observation by the RA, and surveys. For the iEHR+Navigator arm, we will create a log to note the types of CHN outreach efforts, time spent, and the type of services delivered to smoking parents. Personnel costs will reflect national average wages for each personnel type. We will use Monte Carlo simulation methods to develop confidence bounds on our cost-per-quit estimates. Following standard methods of economic evaluation in prior studies, we will also perform parameter-specific sensitivity analyses in which individual parameters are varied singly and in combination, through plausible ranges to assess the relative impact different elements of the program have on overall cost-effectiveness.

3. Assess the implementation and sustainability of the intervention in the iEHR + Navigator arm **NRT prescription, quitline referral, and smokefree homes and cars will be compared between iEHR+Navigator vs. usual care control, as assessed by parental surveys at 12-months, and by EHR documentation data at, before, and immediately following implementation at 6,12, 18, 24, 30, and 36 months (documentation of screening and services delivery fidelity, coding, and billing).** We will follow a similar analysis strategy to that stated above in primary outcome analysis. We will compare cessation assistance delivery at 6 months intervals for 3 years using EHR documentation data. We will analyze qualitative interview data from the combined RE-AIM/CFIR interviews conducted at before and following implementation, and at 12, and 24 months. The interview data will be coded and thematically analyzed in order to gain clarity on implementation processes and intervention sustainability. The interview data will inform future implementation and sustainability efforts.
