# Supplementary material for: Automated Tobacco Cessation Intervention for Parents in Pediatric Primary Care: A Cluster-Randomized Clinical Trial
Source: JAMA Netw Open. 2025 Aug 27;8(8):e2529384. doi: 10.1001/jamanetworkopen.2025.29384 (PMC12391990; doi:10.1001/jamanetworkopen.2025.29384)
Supplement: Supplement 2. — Data Sharing Statement [file jamanetwopen-e2529384-s002.pdf]

Data Sharing Statement

Nabi-Burza. Automated Tobacco Cessation Intervention for Parents in Pediatric Primary Care. *JAMA Netw Open*. Published August 27, 2025. doi:10.1001/jamanetworkopen.2025.29384

Data
Additional Information: ClinicalTrials.gov Identifier: NCT04974736
Data available: Yes
Data types: Deidentified participant data
How to access data: Please email Dr. Jonathan Winickoff with an analysis plan for any data requests.
When available: With publication

Supporting Documents
Document types: None

Additional Information
Who can access the data: Researchers whose proposed use of the data has been approved
Types of analyses: Specified purpose
Mechanisms of data availability: after approval of a proposal and a signed data access agreement
